# Refining the taxonomy of the order Hyphomicrobiales (Rhizobiales) based on whole genome comparisons of over 130 type strains

**DOI:** 10.1099/ijsem.0.006328

**Published:** 2024-04-15

**Authors:** George C. diCenzo, Yuqi Yang, J. Peter W. Young, Nemanja Kuzmanović

**Affiliations:** 1 Department of Biology, Queen's University, Kingston, ON, K7P 0S7, Canada; 2 Department of Biology, University of York, York, YO10 5DD, UK; 3 Institute for Plant Protection in Horticulture and Urban Green, Julius Kühn Institute (JKI) - Federal Research Centre for Cultivated Plants, Braunschweig, 38104, Germany

**Keywords:** *Bartonellaceae*, family boundaries, *Hyphomicrobiales*, *Phyllobacteriaceae*, *Rhizobiales*

## Abstract

The alphaproteobacterial order *Hyphomicrobiales* consists of 38 families comprising at least 152 validly published genera as of January 2024. The order *Hyphomicrobiales* was first described in 1957 and underwent important revisions in 2020. However, we show that several inconsistencies in the taxonomy of this order remain and we argue that there is a need for a consistent framework for defining families within the order. We propose a common genome-based framework for defining families within the order *Hyphomicrobiales*, suggesting that families represent monophyletic groups in core-genome phylogenies that share pairwise average amino acid identity values above ~75 % when calculated from a core set of 59 proteins. Applying this framework, we propose the formation of four new families and to reassign the genera *Salaquimonas*, *Rhodoblastus*, and *Rhodoligotrophos* into *Salaquimonadaceae* fam. nov., *Rhodoblastaceae* fam. nov., and *Rhodoligotrophaceae* fam. nov., respectively, and the genera *Albibacter*, *Chenggangzhangella*, *Hansschlegelia*, and *Methylopila* into *Methylopilaceae* fam. nov. We further propose to unify the families *Bartonellaceae*, *Brucellaceae*, *Phyllobacteriaceae*, and *Notoacmeibacteraceae* as *Bartonellaceae*; the families *Segnochrobactraceae* and *Pseudoxanthobacteraceae* as *Segnochrobactraceae*; the families *Lichenihabitantaceae* and *Lichenibacteriaceae* as *Lichenihabitantaceae*; and the families *Breoghaniaceae* and *Stappiaceae* as *Stappiaceae*. Lastly, we propose to reassign several genera to existing families. Specifically, we propose to reassign the genus *Pseudohoeflea* to the family *Rhizobiaceae*; the genera *Oricola*, *Roseitalea*, and *Oceaniradius* to the family *Ahrensiaceae*; the genus *Limoniibacter* to the emended family *Bartonellaceae*; the genus *Faunimonas* to the family *Afifellaceae*; and the genus *Pseudochelatococcus* to the family *Chelatococcaceae*. Our data also support the recent proposal to reassign the genus *Prosthecomicrobium* to the family *Kaistiaceae*.

## Data summary

Supplementary text is available with the online version of this article. Twelve supplementary figures and 17 supplementary datasets are available through FigShare at https://doi.org/10.6084/m9.figshare.25471522 [1].

## Introduction

The order *Hyphomicrobiales* of the class *Alphaproteobacteria* consists of 38 families encompassing 152 valid genera according to the List of Prokaryotic names with Standing in Nomenclature (LPSN; accessed 2 January 2024). It was known by the name *Rhizobiales* Kuykendall 2006 [2] from 2005 until 2020, when Hördt and colleagues [3] pointed out that this was an illegitimate later synonym of *Hyphomicrobiales* Douglas 1957 (Approved List 1980) [4]. This order contains phenotypically diverse organisms, including terrestrial and aquatic bacteria, plant mutualists, and plant, animal, and human pathogens [2]. Moreover, all known alpha-rhizobia (alphaproteobacterial nitrogen-fixing legume symbionts) are found within the order *Hyphomicrobiales* and are spread across seven families (*Rhizobiaceae*, *Phyllobacteriaceae*, *Brucellaceae*, *Nitrobacteriaceae*, *Methylobacteriaceae*, *Xanthobacteriaceae*, and *Devosiaceae*) [5].

We recently proposed a genome-based framework for defining genera within the family *Rhizobiaceae* [6], which is the largest family within the order *Hyphomicrobiales* with 22 validly published and correct genera as of 2 January 2024. While applying our framework to refine the family *Rhizobiaceae* [6], we wondered whether any genera currently assigned to the family *Rhizobiaceae* should be reassigned to novel families. This prompted us to evaluate family-level classifications within the order *Hyphomicrobiales*. Here, we propose a genome-based framework for defining families within the order *Hyphomicrobiales*. We then apply this framework and propose the unification of several families, the formation of four new families, and the reassignment of multiple genera.

## Methods

### Datasets

Core-proteome phylogenies and overall genomic relatedness indices (OGRIs) were calculated using the genomes of 138 *Hyphomicrobiales* strains (Dataset S1, available through FigShare at https://doi.org/10.6084/m9.figshare.25471522). Where applicable, five *Caulobacterales* strains were included as an outgroup (Dataset S2). Of the 138 *Hyphomicrobiales* strains, 130 were type strains of type species of genera, representing all type strains of type species of *Hyphomicrobiales* genera with publicly available genome sequences at the time of download (June 2023). This included *Sinorhizobium fredii* USDA 205^T^, *Peteryoungia ipomoeae* shin9-1^T^, and *Xaviernesmea oryzae* 505^T^, as we treated *Sinorhizobium*, *Peteryoungia*, and *Xaviernesmea* as correct names despite being marked as synonyms in the LPSN [6,7]. Similarly, we included *Brucella* (syn. *Ochrobactrum*) *anthropi* ATCC 49188^T^ as the status of the genus *Ochrobactrum* is debated [8]. We additionally included *Ancylobacter* (syn. *Angulomicrobium*) *tetraedralis* DSM 5895^T^ and *Ancylobacter* (syn. *Starkeya*) *novellus* ATCC 8093^T^ as the genera *Ancylobacter*, *Angulomicrobium*, and *Starkeya* were not marked as synonyms in the LPSN at the time of download [9]. Likewise, *Afipia* (syn. *Oligotropha*) *carboxidovorans* was included as the genera *Afipia* and *Oligotropha* were not marked as synonyms in the LPSN at the time of download [3]. As the genome sequence of the type strain of the *Hyphomicrobium* type species (*Hyphomicrobium vulgare*) was unavailable, we included the genome of *Hyphomicrobium denitrificans* ATCC 51888^T^ in order to include a representative of the genus *Hyphomicrobium*, as it is the type genus of the order. We additionally included the following four strains as we were particularly interested in the families *Rhizobiaceae* and *Phyllobacteriaceae*: ‘*Pararhizobium mangrovi*’ BGMRC 6574^T^, ‘*Neopararhizobium haloflavum*’ XC0140^T^, ‘*Paramesorhizobium deserti*’ A-3-E^T^, and ‘*Oryzicola mucosus*’ ROOL2^T^. Where noted, we included an additional four species type strains from the genus *Bartonella* and the recently proposed *Flavimaribacter sediminis* WL0058^T^ (Dataset S3). All genomes were downloaded from the National Center for Biotechnology Information (NCBI) genome database.

The following 24 genera are currently assigned to the order *Hyphomicrobiales* but were excluded from the genome-based analyses as whole genome sequences were not publicly available, and/or the genera were not yet validly published, at the time the genomes were downloaded (June 2023): *Albibacter*, *Ancalomicrobium*, *Anderseniella*, *Antarcticirhabdus*, *Blastobacter, Butyratibacter*, *Daeguia*, *Ferranicluibacter*, *Filomicrobium*, *Hansschlegelia*, *Jiella*, *Labrys*, *Lentilitoribacter*, *Mariluticola*, *Methylocella*, *Methylorosula*, *Microbaculum*, *Oryzibacter*, *Pedomicrobium*, *Pinisolibacter*, *Pseudahrensia*, *Psychroglaciecola*, *Seliberia*, and *Youhaiella*. Although the genus *Hyphomicrobium* was represented in the analyses, the type strain of the *Hyphomicrobium* type species, *Hyphomicrobium vulgare* JCM 6889^T^, was not available.

For reconstruction of a 16S rRNA gene phylogeny, full-length 16S rRNA gene sequences were extracted from the genome sequences of the 143 strains used in the genome-based analyses, where available. In cases where more than one distinct 16S rRNA gene sequence was extracted from a whole genome sequence, all unique sequences were kept. If the genome did not contain a full-length 16S rRNA gene sequence, the strain’s 16S rRNA gene sequence was downloaded via links embedded in the LPSN database [10]. This dataset was supplemented with the 16S rRNA gene sequences of all type strains of type species of *Hyphomicrobiales* genera that lacked publicly available whole genome sequences. These 16S rRNA gene sequences were downloaded from the LPSN database (Dataset S4).

### Identification of core gene sets

Initially, the get_homologues software package version 05052023 [11] and get_phylomarkers software package version 2.4.5_17nov2022 [12] were used to identify non-recombining single-copy marker genes present in all 143 genomes, as described previously [13]. This led to the identification of 19 marker genes, which we termed ‘core_143’ (i.e., core genes of the 143 strains). Next, we used get_homologues and custom scripts to identify single-copy marker genes present in at least 95 % of the target genomes; the stringent filtering of get_phylomarkers was not used. This led to the identification of 256 marker genes, which we termed ‘perc95_143’ (i.e., genes found in at least 95 % of the 143 strains). During preliminary investigations of the perc95_143 gene set, we observed that five strains (*Chenggangzhangella methanolivorans* CHL1^T^, *Methylobrevis pamukkalensis* VKM B-2849^T^, *Nitratireductor aquibiodomus* JCM 21793^T^, *Liberibacter crescens* BT-1^T^, and *Methyloligella halotolerans* VKM B-2706^T^) lacked between 12 and 28 % of these genes (see Text S1 of available in the online version of this article). Likewise, the CheckM proteome completeness scores for four of these five strains were below 90%, suggesting they are of low quality. We therefore repeated the above analyses using a reduced set of 138 strains that excluded those five strains. Two additional strains, *Segnochrobactrum spirostomi* Sp-1^T^ and *Ahrensia kielensis* DSM 5890^T^, lacked 9 and 7 % of the perc95_143 genes, respectively; *S. spirostomi* Sp-1^T^ also had a CheckM proteome completeness score of only 88 % but a genome completeness score of 98 %. However, *S. spirostomi* Sp-1^T^ and *A. kielensis* DSM 5890^T^ were kept in the dataset as they were the only representatives of their respective families. All other strains lacked less than 5 % of the perc95_143 genes and had genome and proteome completeness scores >90 % (Dataset S1). Using the reduced dataset of 138 strains, we identified a core set of 59 non-recombining, single-copy marker genes present in all 138 strains (termed ‘core_138’, referring to the core genes of the 138 strains) and 267 single-copy genes present in at least 95 % of the target genomes (termed ‘perc95_138’, referring to genes found in at least 95 % of the 138 strains).

### Calculation of OGRIs

Core-proteome average amino acid identity (cpAAI) was computed as described previously [6], and was calculated as the proportion of differences (including gaps) in pairwise sequence comparisons using the concatenated alignments of the non-recombining, single-copy core marker genes identified using get_phylomarkers (i.e., core_143 or core_138). Calculations were performed using the dist.aa() function of the ‘ape’ package version 5.7–1 in R version 4.3.0 [14].

Whole-proteome average amino acid identity (wpAAI; usually simply known as AAI) was computed using EzAAI version 1.2.2 [15], with default parameters and the dependency Prodigal version 2.6.3 [16].

### Core-proteome phylogenies

For the core_143 and core_138 gene sets, get_phylomarkers was used to align and concatenate the encoded proteins and to remove non-informative sites; alignment was performed using Clustal Omega version 1.2.4 [17]. For the perc95_143 and perc95_138 gene sets, the encoded proteins were aligned with mafft version 7.453 [18], after which the protein alignments were trimmed using trimAl version 1.4.rev22 [19] and the automated1 option, and then concatenated. For all four gene sets, the concatenated protein alignments were used as input for ModelFinder [20] as implemented in iq-tree version 2.2.2.4 [21], and the best scoring model was identified based on Bayesian information criterion (BIC). iq-tree was then used to infer maximum-likelihood (ML) phylogenies from the concatenated alignments using the best-scoring model for each protein set (core_143: LG+F+R8; core_138: LG+F+R9; perc95_143: LG+F+I+R10; perc95_138: LG+F+I+R10). Branch supports were assessed in iq-tree using the Shimodaira–Hasegawa-like approximate likelihood ratio test (SH-aLRT) [22] and ultrafast jackknife analysis with a subsampling proportion of 40%, with both metrics calculated from 1000 replicates. Phylogenies were visualized using iTOL [23].

### 16S rRNA gene phylogenies

The 16S rRNA gene sequences were aligned using Clustal Omega with the --full and --full-iter options, after which the alignment was trimmed with trimAl using the automated1 option. The trimmed nucleotide alignment was used as input for ModelFinder as implemented in iq-tree, which identified GTR+F+I+R6 as the best-scoring model based on the BIC. iq-tree was then used to infer an ML phylogeny using the GTR+F+I+R6 model. Branch supports were assessed in iq-tree, using SH-aLRT and an ultrafast bootstrap analysis, with both metrics calculated from 1000 replicates. We additionally inferred an ML phylogeny using a 16S rRNA gene alignment produced using mafft, but as the outgroup was not monophyletic, this phylogeny was discarded.

### Data availability

All genome sequences used in this work were previously published, and the assembly accessions are provided in Datasets S1–S3. Likewise, all 16S rRNA gene sequences used in this study were previously published, and the corresponding GenBank accessions for those not extracted directly from the whole genome sequences are provided in Dataset S4. Supplementary Texts S1 and S2 are included with the online version of this article. Twelve supplementary figures and 17 supplementary datasets, which includes all raw data (cpAAI data, wpAAI data, and Newick formatted phylogenies) used to generate the figures presented in this manuscript, are available through FigShare https://doi.org/10.6084/m9.figshare.25471522.v1 [24]. All code to repeat the analyses in this study is available through GitHub (https://github.com/diCenzo-Lab/012_2023_Hyphomicrobiales_taxonomy). A bash script, together with the protein marker sets identified in this study, are also available through GitHub to facilitate the application of the proposed taxonomic framework to other organisms.

## Results and discussion

### Genome-based metrics for family-level delimitation in the order *Hyphomicrobiales*

To evaluate family assignments in the order *Hyphomicrobiales*, we began with a dataset of 138 *Hyphomicrobiales* strains, including 130 type strains of type species of genera. The type genera of 36 of the 38 *Hyphomicrobiales* families were represented by the type strain of their type species. Genomes were not available for the type strains of the type species of the type genera of the remaining two families (*Hyphomicrobiales* and *Ancalomicrobium*); these families were instead each represented by at least one other species type strain. Five *Caulobacterales* type strains were included as an outgroup.

**Fig. 1.**
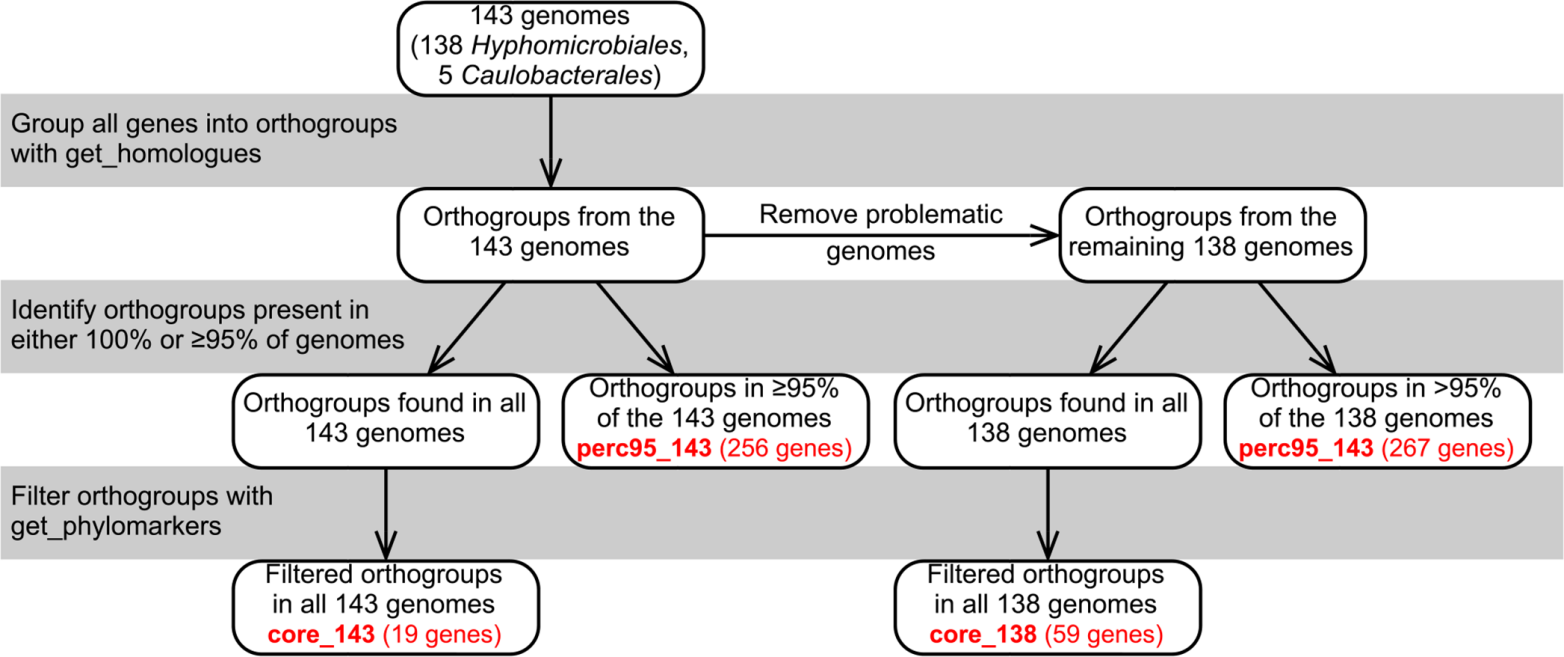
Schematic overview of the identification of the four gene sets used in this study. A simplified overview of the pipeline to identify the four gene sets (core_143, core_138, perc95_143, perc95_138; Table 1) used for phylogenetic and overall genome similarity analyses in this study. After downloading 143 genomes, the get_homologues software was used to group genes into orthogroups. Orthogroups found in all 143 genomes were filtered with get_phylomarkers, resulting in core_143, whereas orthogroups found in at least 95 % of the 143 genes were taken as perc95_143. In addition, genes from five problematic genomes were removed from the original set of orthogroups, leaving orthogroups corresponding to the remaining 138 genomes. Orthogroups found in all 138 genomes were filtered with get_phylomarkers, resulting in core_138, whereas orthogroups found in at least 95 % of the 138 genomes were taken as perc95_138.

**Fig. 2.**
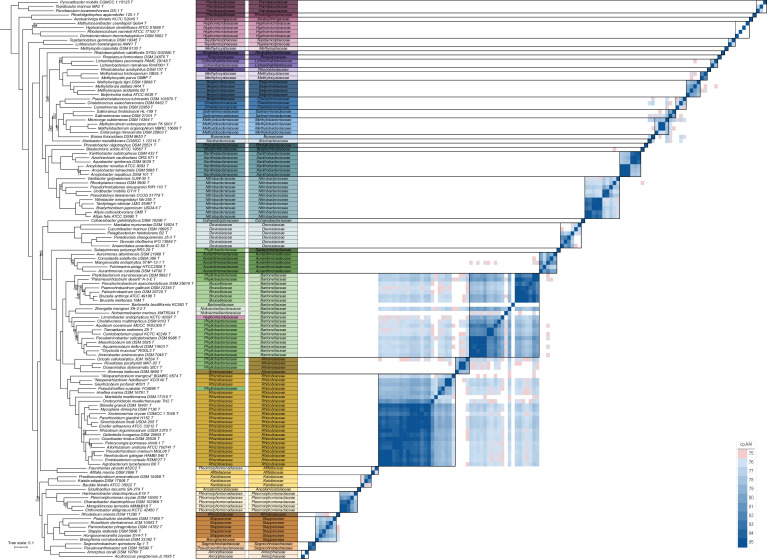
Phylogenetic and core-proteome AAI (cpAA) analyses of the order *Hyphomicrobiales*. On the left, a maximum likelihood phylogeny of 133 *Hyphomicrobiales* type strains is shown, built using the concatenated protein alignments encoded by the perc95_138 gene set (267 genes present in at least 95 % of the strains). The phylogeny was rooted using five *Caulobacterales* type strains as the outgroup. The numbers on the nodes indicate the ultra-fast jackknife values using a 40 % resampling rate (top numbers) and the SH-aLRT support values (bottom numbers), both calculated from 1000 replicates. Values are only shown at nodes where at least one value is below 100. The scale bar represents the average number of amino acid substitutions per site. To the right of the phylogeny is the current family assignment of each of the 133 *Hyphomicrobiales* type strains, followed to the right by the proposed family assignment of each strain. On the right-hand side, a matrix is provided showing the cpAAI values between each pair of strains calculated using the proteins encoded by the core_138 gene set (59 genes present in 100 % of the strains). Values less than 75 % are in white while all values greater than 85 % are the same shade of blue. Black boxes indicate the proposed families.

As in our recent study of the family *Rhizobiaceae* [6], we reasoned that genome sequence-based family delineation should consider both phylogenetic relatedness and genome similarity based on one or more OGRIs. We chose to work with cpAAI and wpAAI as these appeared to be the most appropriate OGRIs based on our previous work [6]. Therefore, we first identified a set of 19 marker genes present in 100 % of the 143 strains (termed core_143) and a set of 256 marker genes present in ≥95 % of the strains (termed perc95_143). In addition, using a reduced dataset of 138 strains (see the Methods section and Text S1 of Supplementary Material), we identified a set of 59 marker genes present in all 138strains (termed core_138) and a set of 267 marker genes present in ≥95 % of the 138 strains (termed perc95_138). A summary of the four gene sets is provided in Table 1, and a graphical representation of how they were identified is given as Fig. 1. All four datasets were used to reconstruct ML phylogenies (Figs 2 and S1–S3), and core_143 and core_138 were additionally used to calculate pairwise cpAAI values between all 143 or 138 strains, respectively (Figs 2 and S1, Datasets S5 and S6). Pairwise wpAAI was also calculated between all 143 strains (Fig. S4, Dataset S7).

**Table 1.** Properties of the gene sets used for phylogenetic and overall genome similarity analyses

	Gene set			
	core_143	perc95_143	core_138	perc95_138
Number of *Hyphomicrobiales* strains	138	138	133	133
Number of *Caulobacterales* strains	5	5	5	5
Genomes containing the genes (%)	100	≥95	100	≥95
Number of genes	19	256	59	267

**Fig. 3.**
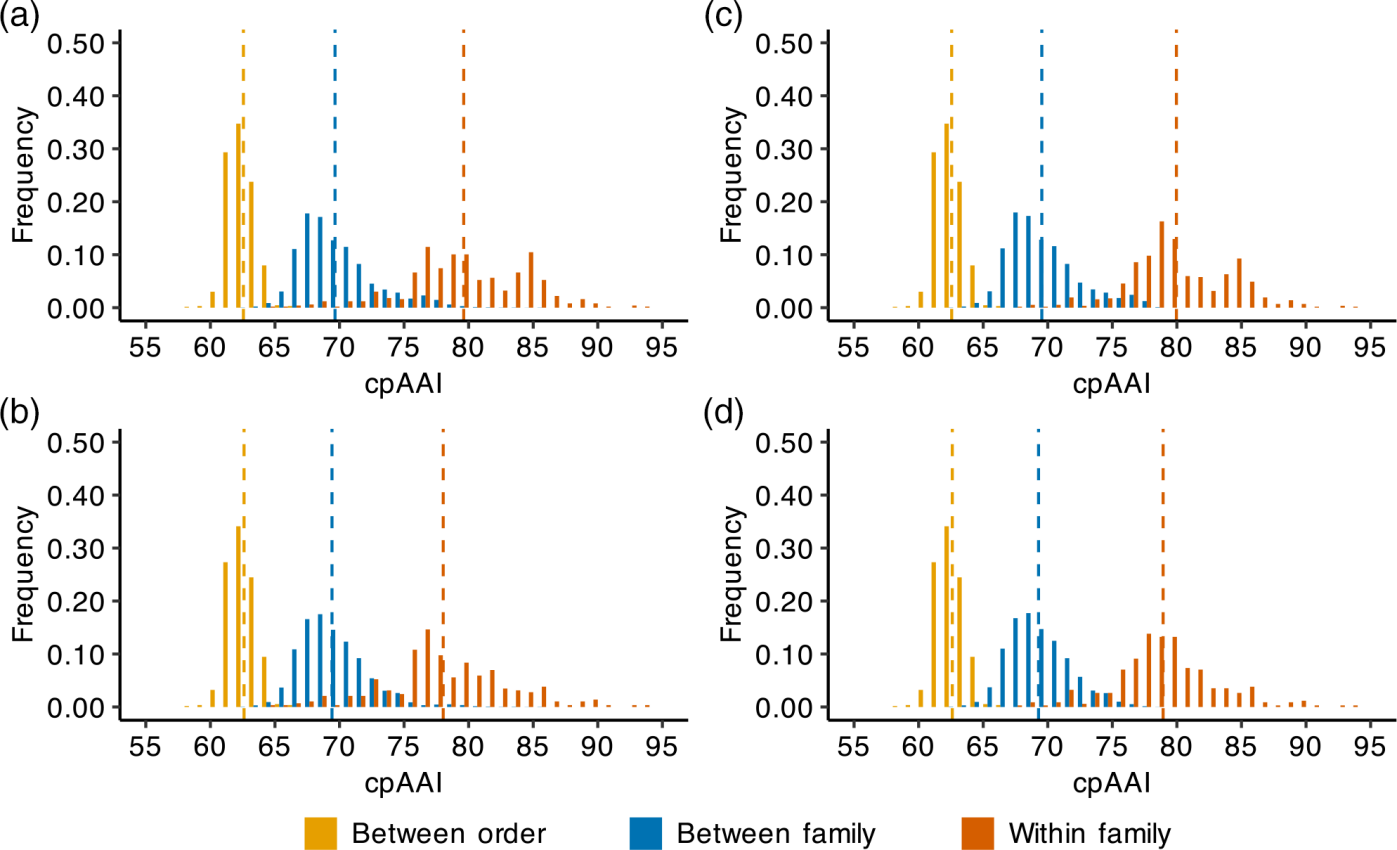
Distribution of core-proteome AAI (cpAAI) comparisons of the order *Hyphomicrobiales*. Pairwise cpAAI values were calculated based on 59 nonrecombinant loci from the core genome of 133 members of the order *Hyphomicrobiales* and five members of the order *Caulobacterales*. Results are summarized as histograms with a bin width of 1 %. The cpAAI values calculated between strains belonging to different orders (yellow; i.e., between *Hyphomicrobiales* strains and strains of the *Caulobacterales* outgroup), different families of the order *Hyphomicrobiales* (blue), or the same family of the order *Hyphomicrobiales* (red) are summarized separately. Dashed vertical lines represent the mean value of each distribution. In all plots, cpAAI values where both strains belong to the order *Caulobacterales* were excluded. (**a**) The distribution of all pairwise cpAAI values with the classification (i.e., between order, between family, or within family) based on existing taxonomic assignments. (**b**) The distribution of all pairwise cpAAI values except for those including at least one strain from the family *Rhizobiaceae*, with the classification based on existing taxonomic assignments. (**c**) The distribution of all pairwise cpAAI values with the classification based on the proposed taxonomic assignments. (**d**) The distribution of all pairwise cpAAI values except for those including at least one strain from the family *Rhizobiaceae*, with the classification based on the proposed taxonomic assignments.

We wondered whether we could identify biologically relevant cpAAI or wpAAI thresholds for delineating families in the order *Hyphomicrobiales*. We therefore plotted a histogram of the distribution of cpAAI values (calculated from core_138), plotting separate distributions for within-family (i.e., between strains of the same *Hyphomicrobiales* family), between-family but within-order (i.e., between strains of different *Hyphomicrobiales* families), and between-order (i.e., between *Hyphomicrobiales* strains and strains of the *Caulobacterales* outgroup) comparisons (Fig. 3a, c). In addition to using all cpAAI values calculated from core_138, we plotted the results for a reduced dataset lacking the family *Rhizobiaceae* (Fig. 3b, d). This was done for two reasons. The family *Rhizobiaceae* was over-represented in our dataset, accounting for over 15 % of the strains in our analysis, and thus its inclusion had the potential to bias the distribution. In addition, the family *Rhizobiaceae* was not well separated from the related *Bartonellaceae–Phyllobacteriaceae–Brucellaceae–Notoacmeibacteraceae* clade (as discussed below), and thus had the potential to mask patterns that might otherwise be detected across the remaining 33 *Hyphomicrobiales* families.

The within-family, between-family, and between-order distributions of cpAAI were clearly distinct, with mean values of 78.0 % [standard deviation (SD) 4.7 %], 69.4 % (SD 2.6 %), and 62.6 % (SD 1.0 %), respectively, for the dataset lacking the family *Rhizobiaceae*; the differences between all distributions were statistically significant as determined by a Kruskal–Wallis test followed by pairwise Wilcoxon rank sum tests (p-value < 2.2e-16). Notably, excluding the family *Rhizobiaceae*, 97.1 % of the between-family comparisons fell below 75%, while 80.1 % of the within-family comparisons were above 75 % (Fig. 3b). When considering the taxonomic changes proposed below, this increases to 98.3 % of between-family comparisons being below 75 and 88.5 % (93.8 % when excluding *Bartonella bacilliformis* KC583^T^ that has a highly reduced genome) of within-family comparisons being above 75 % (Fig. 3d). These results, together with the shapes of the distributions, suggested to us that a cpAAI value of ~75 %, when calculated from core_138, represents a natural threshold for defining families within the order *Hyphomicrobiales*. Performing similar analyses using cpAAI values calculated from core_143 (Fig. S5), or the wpAAI data (Fig. S6), also allowed for the identification of potential thresholds (~78 and ~63 %, respectively) for defining families within the order *Hyphomicrobiales*. However, these thresholds were less well defined compared to the threshold identified for core_138 and thus we focus our analyses primarily on the cpAAI data generated with core_138.

We attempted to supplement our genome-based analyses with 16S rRNA gene phylogenetic studies (Fig. S7). However, our 16S rRNA gene phylogeny was non-concordant with the genome-based phylogenies and failed to accurately resolve multiple families, consistent with past studies [25]. We therefore only made use of the 16S rRNA gene phylogeny for taxa lacking whole genome sequencing data.

In the following sections, we propose several taxonomic revisions in the order *Hyphomicrobiales* based on overall genomic similarity and the need for families to be monophyletic. In general, we aimed to be conservative and limit the number of changes that we propose, and therefore have not proposed to split monophyletic families based solely on low cpAAI values. We do, however, propose to unify sister families where appropriate and when clearly supported by the cpAAI data.

### Taxonomic implications for the families *Rhizobiaceae*, *Ahrensiaceae*, *Bartonellaceae*, *Brucellaceae*, *Phyllobacteriaceae*, and *Notoacmeibacteraceae*

The six families *Rhizobiaceae*, *Ahrensiaceae*, *Bartonellaceae*, *Brucellaceae*, *Phyllobacteriaceae*, and *Notoacmeibacteraceae* collectively represent over a third of the *Hyphomicrobiales* strains included in our analyses. Of note was the family *Phyllobacteriaceae*, which was not monophyletic in any of the phylogenies and whose type strain of the type species of the genus (*Phyllobacterium myrsinacearum* DSM 5892^T^) consistently formed its own lineage separate from all other members of the family (Figs 2 and S1–S3). *Pseudohoeflea suaedae* YC6898^T^ was consistently nested within the family *Rhizobiaceae* in all phylogenies (Figs 2 and S1–S3). We therefore propose that the genus *Pseudohoeflea* be reassigned to the family *Rhizobiaceae*. In addition, *Salaquimonas pukyongi* RR3-28^T^ consistently formed its own lineage (Figs 2 and S1–S3), and when calculated from core_138, displayed pairwise cpAAI values <74 % against all other strains (Fig. 2). We therefore propose that the genus *Salaquimonas* be reassigned to the family *Salaquimonadaceae* fam. nov. Moreover, *Oricola cellulosilytica* JCM 19534^T^, *Roseitalea porphyridii* MA7-20^T^, and *Oceaniradius stylonematis* StC1^T^ consistently grouped separately from the rest of the family *Phyllobacteriaceae* and instead formed a clade with *A. kielensis* DSM 5890^T^ (Figs 2 and S1–S3), the type strain for the type genus of the family *Ahrensiaceae*. When calculated from core_138, all pairwise cpAAI values between the four strains were >78 % (Fig. 2). We therefore propose that the genera *Oricola*, *Roseitalea*, and *Oceaniradius* be reassigned to the family *Ahrensiaceae*.

The remaining 11 members of the family *Phyllobacteriaceae* included in this analysis were paraphyletic in all four phylogenies (Figs 2 and S1–S3). The minimal monophyletic group containing all 11 *Phyllobacteriaceae* species also includes the families *Brucellaceae*, *Bartonellaceae*, and *Notoacmeibacteraceae*; the inclusion of the genus *Bartonella* within this monophyletic group was further supported by supplemental phylogenetic analyses (see Text S2 of Supplementary Material; Figs S8–S11). As a result, it was necessary either to split the family *Phyllobacteriaceae* into three families or to unify the families *Phyllobacteriaceae*, *Brucellaceae*, *Bartonellaceae*, and *Notoacmeibacteraceae*. The cpAAI and wpAAI measurements favour the latter option; when calculated from core_138, all pairwise cpAAI values between members of these families were above 76.5 %, except for comparisons involving *B. bacilliformis* KC583^T^, which has a highly reduced genome (cpAAI values involving this strain ranged from 69–74.5 %), or *Notoacmeibacter marinus* XMTR2A4^T^, which displayed unexpectedly low cpAAI values against all strains (cpAAI values involving this strain ranged from 69–76 %). Likewise, most cpAAI comparisons calculated with core_143 and wpAAI values were above the proposed thresholds of 78 and 63 %, respectively. Based on these results, we propose that the families *Bartonellaceae*, *Brucellaceae*, *Notoacmeibacteraceae*, and *Phyllobacteriaceae* be unified with the name *Bartonellaceae* as it takes priority. In addition, the strain *Limoniibacter endophyticus* KCTC 42097^T^, currently assigned to the family *Hyphomicrobiaceae*, was consistently nested within the emended family *Bartonellaceae* in all phylogenies. We therefore propose that the genus *Limoniibacter* be reassigned to the family *Bartonellaceae*.

In contrast to our initial expectations [6], the data suggested that the family *Rhizobiaceae* did not need to be split into two or more families, while our supplemental phylogenetic analyses (see Text S2 of Supplementary Material; Figs S8–S11) supported the inclusion of the recently proposed genus *Flavimaribacter* in the family *Rhizobiaceae* [26]. On the other hand, when calculated from core_138, 82.5 % of the cpAAI comparisons between members of the family *Rhizobiaceae* and the emended family *Bartonellaceae* were above our proposed threshold of 75 % (Fig. 2). Similarly, 71.2 % of the wpAAI comparisons between members of the family *Rhizobiaceae* and the emended family *Bartonellaceae* were above our proposed threshold of 63 % (Fig. S4), although only 36.6 % of the cpAAI comparisons were above our proposed threshold of 78 % when calculated from core_143 (Fig. S1). The possibility of uniting these two families therefore merits consideration. However, we do not favour this approach for two reasons. First, the between-family cpAAI values (calculated from core_138) tended to be lower than the within-family cpAAI values (Fig. 2), with average values of 75.9 % (SD 1.7  %) and 80.5 % (SD 3.8 %), respectively (p-value < 2.2e16 based on a Wilcoxon rank sum test); qualitatively similar results are seen with cpAAI calculated from core_143 and the wpAAI data (Figs S1 and S4). In addition, in three of the four phylogenies, the families *Rhizobiaceae* and *Bartonellaceae* were not sister taxa; instead, these families only formed a monophyletic group when the emended family *Ahrensiaceae* was also included. The family *Ahrensiaceae* was better delineated from the family *Rhizobiaceae* based on cpAAI and wpAAI values (Figs 2, S1, and S4); the average cpAAI value (when calculated from core_138) for comparisons between species of the families *Ahrensiaceae* and *Rhizobiaceae* was 74.1%, with 84 % of comparisons below the proposed threshold of ~75 % for family delineation. Likewise, in the cases of cpAAI calculated from core_143 and wpAAI, 99 and 86 %, respectively, of the comparisons between species of the families *Ahrensiaceae* and *Rhizobiaceae* were below the proposed thresholds for family delineation. These results suggest it would not be appropriate to unify the families *Ahrensiaceae* and *Rhizobiaceae*. Considering the weight of the evidence, we have therefore chosen to leave the family *Rhizobiaceae*, the emended family *Ahrensiaceae*, and the emended family *Bartonellaceae* as distinct families.

### Taxonomic implications: formation of new families

Several families in the order *Hyphomicrobiales* were paraphyletic in all four of our phylogenies. To fix the paraphyly and ensure all families are monophyletic, we propose the formation of three new families and the reassignment of two genera to other existing families (see the following section).

The type strain *Rhodoblastus acidophilus* DSM 137^T^ is currently assigned to the family *Roseiarcaceae*. However, *R. acidophilus* DSM 137^T^ did not form a monophyletic group with the type strain *Roseiarcus fermentans* DSM 24875^T^ that represents the type genus of the family *Roseiarcaceae*. Instead, *R. acidophilus* DSM 137^T^ formed its own lineage as a sister taxon to the family *Methylocystaceae* in all four phylogenies (Figs 2 and S1–S3). When calculated from core_138, the pairwise cpAAI values between *R. acidophilus* DSM 137^T^ and two type strains of the family *Methylocystaceae* were 74.8 and 74.5 % (Fig. 2), which are below our proposed threshold of 75 % for family delimitation. We therefore propose to reassign the genus *Rhodoblastus* to the family *Rhodoblastaceae* fam. nov.

The genus *Methylopila* was not assigned to a family when it was proposed [27], but was subsequently assigned to the family *Methylocystaceae* [28]. However, the type strain *Methylopila capsulata* DSM 6130^T^ did not cluster with the other members of the family *Methylocystaceae*, and instead formed its own distinct lineage in the two phylogenies based on core_138 or perc95_138 (Figs 2 and S4). When calculated from core_138, all comparisons involving *M. capsulata* DSM 6130^T^ gave cpAAI values less than 74 % (Fig. 2). In the phylogenies based on core_143 or perc95_143, which include five additional type strains, *M. capsulata* DSM 6130^T^ formed a clade with *C. methanolivorans* CHL1^T^ also currently assigned to the family *Methylocystaceae* (Figs S1 and S2). When calculated from core_143, these two strains had a pairwise cpAAI value of 86.5 % (Fig. S1), which is above the proposed threshold of 78 % for family delineation. The 16S rRNA gene phylogeny (Fig. S7) included two additional type strains, *Hansschlegelia plantiphila* VKM B-2347^T^ and *Albibacter methylovorans* DM10^T^, of genera currently assigned to the family *Methylocystaceae* but that lack publicly available whole genome sequence. In the 16S rRNA phylogeny, *H. plantiphila* VKM B-2347^T^ and *A. methylovorans* DM10^T^ formed a monophyletic clade with *M. capsulata* DSM 6130^T^ and *C. methanolivorans* CHL1^T^, rather than with *Methylocystis parva* OBBP^T^, the type strain of the type genus of the family *Methylocystaceae*. Based on these results, we propose to reassign the genera *Methylopila*, *Chenggangzhangella*, *Hansschlegelia*, and *Albibacter* to the family *Methylopilaceae* fam. nov.

The type strain *Rhodoligotrophos appendicifer* 120-1^T^ is currently assigned to the family *Parvibaculaceae*. However, instead of clustering with the other members of the family *Parvibaculaceae*, it formed its own lineage as a sister taxon to the type strain *Aestuariivirga litoralis* KCTC 52945^T^ of the family *Aestuariivirgaceae* in all four phylogenies (Figs 2 and S1–S3). When calculated from core_138, the pairwise cpAAI value between *R. appendicifer* 120-1^T^ and *A. litoralis* KCTC 52945^T^ was 71.2 % (Fig. 2), which is below our proposed threshold of 75 % for family delineation. We therefore propose to reassign the genus *Rhodoligotrophos* to the family *Rhodoligotrophaceae* fam. nov.

### Taxonomic implications: reassignment of genera

*Faunimonas pinastri* A52C2^T^, the type strain of the type species of the genus *Faunimonas*, is currently assigned to the family *Pleomorphomonadaceae* but did not cluster with other members of this family. Instead, in all four phylogenies, *F. pinastri* A52C2^T^ formed a clade with the type strain *Afifella marina* DSM2698^T^ that represents the only genus of the family *Afifellaceae* (Figs 2 and S1–S3). When calculated from core_138, the cpAAI value between *F. pinastri* A52C2^T^ and *A. marina* DSM2698^T^ was 75.6 % (Fig. 2), which is above our proposed threshold of 75 % for family delimitation. We therefore propose to reassign the genus *Faunimonas* to the family *Afifellaceae*.

The genus *Pseudochelatococcus* was not assigned to a family when it was proposed [29], but was subsequently associated with the family *Beijerinckiaceae* [30]. However, the type strain *Pseudochelatococcus lubricantis* DSM 103870^T^ did not cluster with the other members of the family *Beijerinckiaceae*, and instead formed a clade with *Chelatococcus asaccharovorans* DSM 6462^T^ and *Camelimonas lactis* DSM22958^T^ from the family *Chelatococcaceae* in all four phylogenies (Figs 2 and S1–S3). Moreover, the family *Chelatococcaceae* was not monophyletic without the inclusion of *P. lubricantis* DSM 103870^T^ (Figs 2 and S1–S3). When calculated from core_138, all pairwise cpAAI values in this clade were >75 % (Fig. 2). We therefore propose to assign the genus *Pseudochelatococcus* to the family *Chelatococcaceae*.

*Prosthecomicrobium pneumaticum* DSM 16268^T^, the type strain of the type species of the genus *Prosthecomicrobium*, was previously assigned to the family *Hyphomicrobiaceae* [31], although it was recently proposed that the genus *Prosthecomicrobium* be reassigned to the family *Kaistiaceae* [32]. In all four phylogenies, *P. pneumaticum* DSM 16268^T^ formed a clade with *Kaistia adipata* DSM 17808^T^ and *Bauldia litoralis* ATCC 35022^T^ of the family *Kaistiaceae* rather than with species of the family *Hyphomicrobiaceae* (Figs 2 and S1–S3). Moreover, the family *Kaistiaceae* was not monophyletic without the inclusion of *P. pneumaticum* DSM 16268^T^ (Figs 2 and S1–S3). When calculated from core_138, all pairwise cpAAI values in this clade were >75 % (Fig. 2). Our data are therefore consistent with the proposal to reassign the genus *Prosthecomicrobium* to the family *Kaistiaceae*.

### Taxonomic implications: unification of families

The families *Segnochrobactraceae* and *Pseudoxanthobacteraceae* each contain a single validly published genus. The type strains of the type species of these two genera formed a monophyletic group in all four phylogenies (Figs 2 and S1–S3). When comparing the genomes of these two strains, the pairwise cpAAI value was 82.9 % when calculated from core_138 (Fig. 2) and 85.9 % when calculated from core_143 (Fig. S1), while the wpAAI value was 70.0 % (Fig. S4). Given that these values are all well above the proposed thresholds for family delimitation, we propose that the families *Segnochrobactraceae* and *Pseudoxanthobacteraceae* be unified with the name *Segnochrobactraceae* as it takes priority.

The families *Lichenihabitantaceae* and *Lichenibacteriaceae* each contain a single validly published genus, based on strains that were originally isolated from lichens in the Antarctic and the subarctic zone of the northern hemisphere, respectively [33,34]. The type strains of the type species of these two genera formed a monophyletic group in all four phylogenies (Figs 2 and S1–S3). When comparing the genomes of these two strains, the pairwise cpAAI value was 77.9 % when calculated from core_138 (Fig. 2) and 78.9 % when calculated from core_143 (Fig. S1), while the wpAAI value was 67 % (Fig. S4). Given that these values are all above the proposed thresholds for family delimitation, we propose that the families *Lichenihabitantaceae* and *Lichenibacteriaceae* be unified with the name *Lichenihabitantaceae* as it takes priority.

The family *Breoghaniaceae* contains a single genus while the family *Stappiaceae* contains five genera. The six type strains from these two families formed a monophyletic group in all four phylogenies (Figs 2 and S1–S3). When calculated from core_138, the pairwise cpAAI values between members of the family *Stappiaceae* ranged between 77.0 and 81.6 %, which overlapped the cpAAI range of 76.2–78.9 % for comparisons between members of the family *Stappiaceae* and *Breoghania corrubedonensis* DSM 23382^T^ (Fig. 2). Similar results are seen when using cpAAI calculated from core_143 and wpAAI values (Figs 1 and S4). Considering these results, we propose that the families *Stappiaceae* and *Breoghaniaceae* be unified with the name *Stappiaceae*. Both family names were published in the same article [3] but *Stappiaceae* is our preferred choice because *Breoghaniaceae* includes only a single genus.

### Other taxonomic implications

In this study, we refrained from splitting monophyletic families based solely on cpAAI data. Nevertheless, multiple families merit further study as candidates for splitting, namely the families *Amorphaceae*, *Devosiaceae*, *Hyphomicrobiaceae*, and *Parvibaculaceae* (Fig. 2). Additionally, a couple of clades were not well-resolved based on the cpAAI data and the proposed thresholds, and thus also merit further study. The family *Rhodobiaceae* and the emended family *Stappiaceae* are sister taxa that are not well separated in the cpAAI data calculated from core_138 (Fig. 2), with four of the six between-family comparisons above 75 %, although better separation is observed in the cpAAI data calculated from core_143 and the wpAAI data (Figs S1 and S4). Likewise, the families *Methylobacteriaceae* and *Salinarimonadaceae* were not well separated by the cpAAI or wpAAI data; six of the eight cpAAI values calculated from core_138 were above 75 % with the other two above 74.7 % (Fig. 2), four of the eight cpAAI values calculated from core_143 were above 78 % with the other four above 77.5 % (Fig. S1), and all wpAAI values were above the proposed threshold of 63 % (Fig. S4). As there was not overwhelming support for unification of the families *Rhodobiaceae* and *Stappiaceae*, or the families *Methylobacteriaceae* and *Salinarimonadaceae*, we chose to leave all four as distinct families. However, these taxa merit further consideration in future work.

Although it is not a type strain of a type species of a genus, we included the species type strain ‘*P. mangrovi*’ BGMRC 6574^T^ in our dataset as preliminary investigations of the family *Rhizobiaceae* suggested it belonged to a novel genus and potentially a novel family. Our full analysis did not support the reassignment of ‘*P. mangrovi*’ BGMRC 6574^T^ to a new family but it did support the recent proposal to reclassify this strain as ‘*Allopararhizobium mangrovi*’ BGMRC 6574^T^ once validly published [35].

### Conclusions and future directions

We propose a taxonomic framework in which families of the order *Hyphomicrobiales* are defined as monophyletic groups that share pairwise average amino acid identity values above ~75 % when calculated from a core set of 59 proteins (i.e., protein set core_138). Based on this taxonomic framework, we propose to unify the families *Bartonellaceae*, *Brucellaceae*, *Phyllobacteriaceae*, and *Notoacmeibacteraceae* as *Bartonellaceae* (emended description provided below); the families *Segnochrobactraceae* and *Pseudoxanthobacteraceae* as *Segnochrobactraceae*; the families *Lichenihabitantaceae* and *Lichenibacteriaceae* as *Lichenihabitantaceae*; and the families *Breoghaniaceae* and *Stappiaceae* as *Stappiaceae*. We additionally propose the formation of four new *Hyphomicrobiales* families (*Salaquimonadaceae* fam. nov., *Rhodoblastaceae* fam. nov., *Rhodoligotrophaceae* fam. nov., and *Methylopilaceae* fam. nov.), whose formal descriptions are provided below. Lastly, seven other genera are reassigned to other existing families. Our data also support the transfer of the genus *Prosthecomicrobium* to the family *Kaistiaceae*, which was recently proposed [32] but not yet reflected in the LPSN. A summary of taxonomic changes proposed in this study is provided as Table 2.

**Table 2.** Summary of the proposed taxonomic revisions

**Family**	Genera after proposed revisions	Genera added	Genera removed
**Proposed families**			
*Methylopilaceae* fam. nov.	*Alibibacter*, *Cheggangzhangella*, *Hansschlegelia*, *Methylopila* (the type genus)	*Alibibacter*, *Cheggangzhangella*, *Hansschlegelia*, *Methylopila*	–
*Rhodoblastaceae* fam. nov.	*Rhodoblastus* (the type genus)	*Rhodoblastus*	–
*Rhodoligotrophaceae* fam. nov.	*Rhodoligotrophos* (the type genus)	*Rhodoligotrophos*	–
*Salaquimonadaceae* fam. nov.	*Salaquimonas* (the type genus)	*Salaquimonas*	–
**Families removed as a result of unification**			
*Breoghaniaceae*	None	–	*Breoghania*
*Brucellaceae*	None	–	*Brucella*, *Daeguia*, *Falsochrobactrum*, *Ochrobactrum*, *Paenochrobactrum*, *Pseudochrobactrum*
*Lichenibacteriaceae*	None	–	*Lichenibacterium*
*Notoacmeibacteraceae*	None	–	*Notoacmeibacter*, *Zhengella*
*Phyllobacteriaceae*	None	–	*Aminobacter*, *Aquamicrobium*, *Aquibium*, *Chelativorans*, *Corticibacterium*, *Mesorhizobium*, *Nitratireductor*, *Oceaniradius*, *Oricola*, *Phyllobacterium*, *Pseudaminobacter*, *Pseudohoeflea*, *Roseitalea*, *Salaquimonas*, *Tianweitania*
*Pseudoxanthobacteraceae*	None	–	*Pseudoxanthobacter*
**Families with changes in the assigned genera**			
*Afifellaceae*	*Afifella* (the type genus), *Faunimonas*	*Faunimonas*	–
*Ahrensiaceae*	*Ahrensia* (the type genus), *Pseudahrensia*, *Oricola*, *Roseitalea*, *Oceaniradius*	*Oricola*, *Roseitalea*, *Oceaniradius*	–
*Bartonellaceae*	*Aminobacter*, *Aquamicrobium*, *Aquibium*, *Bartonella* (the type genus), *Brucella*, *Chelativorans*, *Corticibacterium*, *Daeguia*, *Falsochrobactrum*, *Limoniibacter*, *Mesorhizobium*, *Nitratireductor*, *Notoacmeibacter*, *Ochrobactrum*, *Paenochrobactrum*, *Phyllobacterium*, *Pseudaminobacter*, *Pseudochrobactrum*, *Tianweitania*, *Zhengella*	*Aminobacter*, *Aquamicrobium*, *Aquibium*, *Brucella*, *Chelativorans*, *Corticibacterium*, *Daeguia*, *Falsochrobactrum*, *Limoniibacter*, *Mesorhizobium*, *Nitratireductor*, *Notoacmeibacter*, *Ochrobactrum*, *Paenochrobactrum*, *Phyllobacterium*, *Pseudaminobacter*, *Pseudochrobactrum*, *Tianweitania*, *Zhengella*	–
*Beijerinckiaceae*	*Beijerinckia* (the type genus), *Methylocapsa*, *Methylocella*, *Methyloferula*, *Methylorosula*, *Methylovirgula*	–	*Pseudochelatococcus*
*Chelatococcaceae*	*Camelimonas*, *Chelatococcus* (the type genus), *Pseudochelatococcus*	*Pseudochelatococcus*	–
*Hyphomicrobiaceae*	*Dichotomicrobium*, *Filomicrobium*, *Hyphomicrobium* (the type genus), *Methyloceanibacter*, *Methyloligella*, *Pedomicrobium*, *Rhodomicrobium*, *Seliberia*	–	*Limoniibacter*
*Lichenihabitantaceae*	*Lichenibacterium*, *Lichenihabitans* (the type genus)	*Lichenibacterium*	–
*Methylocystaceae*	*Methylocystis* (the type genus), *Methylosinus*	–	*Alibibacter*, *Cheggangzhangella*, *Hansschlegelia*, *Methylopila*
*Parvibaculaceae*	*Parvibaculum* (the type genus), *Tepidicaulis*	–	*Rhodoligotrophos*
*Pleomorphomonadaceae*	*Chthonobacter*, *Hartmannibacter*, *Methylobrevis*, *Mongoliimonas*, *Oharaeibacter*, *Pleomorphomonas* (the type genus)	–	*Faunimonas*
*Rhizobiaceae*	*Agrobacterium*, *Allorhizobium*, *Ciceribacter*, *Endobacterium*, *Ensifer*, *Ferranicluibacter*, *Flavimaribacter, Gellertiella*, *Georhizobium*, *Hoeflea*, *Lentilitoribacter*, *Liberibacter*, *Martelella*, *Mycoplana*, *Neorhizobium*, *Onobrychidicola*, *Pararhizobium*, *Peteryoungia*, *Pseudohoeflea*, *Pseudorhizobium*, *Rhizobium* (the type genus), *Shinella*, *Sinorhizobium*, and *Xaviernesmea*	*Pseudohoeflea*	–
*Roseiarcaceae*	*Roseiarcus* (the type genus)	–	*Rhodoblastus*
*Segnochrobacteraceae*	*Pseudoxanthobacter*, *Segnochrobactrum* (the type genus)	*Segnochrobactrum*	–
*Stappiaceae*	*Breoghania*, *Hongsoonwoonella*, *Pannonibacter*, *Pseudovibrio*, *Roseibium*, *Stappia* (the type genus)	*Breoghania*	–

To facilitate the use of our proposed taxonomic framework for assigning new genera to the correct family in the order *Hyphomicrobiales*, we have made an easy-to-use pipeline and the proteins encoded by all gene sets (core_143, core_138, perc95_143, perc95_138) freely available via GitHub (https://github.com/diCenzo-Lab/012_2023_Hyphomicrobiales_taxonomy/tree/main/3_Pipeline). The user provides their genome(s) of interest (as a nucleotide fasta file) and the location of the gene sets as input. The pipeline will then identify orthologs of the core_138 gene set in the new genome(s) and calculate pairwise cpAAI values between the new genomes and all genomes included in our current study. It will additionally identify orthologs of the perc95_138 gene set in the new genome(s) and produce an ML phylogeny (with the LG+F+I+R10 model, identified as the best scoring model for this dataset in our study) that includes the new genome(s) and the 138 genomes included in the phylogeny of Fig. 2. To demonstrate the utility of this pipeline, we applied it to classify five type strains with recently available genome sequences not included in our original analyses (*Antarcticirhabdus aurantiaca* R10^T^, *Ferranicluibacter rubi* CRRU44^T^, *Mariluticola halotolerans* LMO-2^T^, *Oryzibacter oryziterrae* COJ-58^T^, and *Youhaiella tibetensis* CGMCC 1.12719^T^). All five type strains were assigned to the expected family (Fig. S12).

Currently, publicly available whole genome sequences of 20 type strains of type species of *Hyphomicrobiales* genera are not available (see Methods). We encourage researchers interested in these taxa to obtain genome sequences of these type strains to ensure the corresponding genera are assigned to the correct families.

## Description of *Methylopilaceae* fam. nov.

*Methylopilaceae* (Me.thyl.o.pi.la’ce.ae. N.L. fem. n. *Methylopila*, type genus of the family; -*aceae*, ending to denote a family; N.L. fem. pl. n. *Methylopilaceae*, the *Methylopila* family).

Cells are aerobic, Gram-negative, rod-shaped or cocci, and motile or non-motile. The G+C content as calculated from genome sequences is 68.2–69.7 mol%, while the range provided in the literature is 64.4–70.4 mol% [36,37]. The family currently comprises the genera *Albibacter*, *Chenggangzhangella*, *Hansschlegelia*, and *Methylopila* (the type genus). This family can be differentiated from other families based on phylogenetic analyses of core proteome and 16S rRNA gene sequences, as well as OGRI calculations (cpAAI and wpAAI).

## Description of *Rhodoblastaceae* fam. nov.

*Rhodoblastaceae* (Rho.do.blast.a’ce.ae. N.L. masc. n. *Rhodoblastus*, type genus of the family; -*aceae*, ending to denote a family; N.L. fem. pl. n. *Rhodoblastaceae*, the *Rhodoblastus* family).

The description is as given for *Rhodoblastus* [38], which is the type and currently the sole genus of the family. This family can be differentiated from other families based on phylogenetic analyses of core proteome sequences and OGRI calculations (cpAAI and wpAAI).

## Description of *Rhodoligotrophaceae* fam. nov.

*Rhodoligotrophaceae* (Rho.do.li.go.tro’ph.a’ce.ae. N.L. masc. n. *Rhodoligotrophos*, type genus of the family; -*aceae*, ending to denote a family; N.L. fem. pl. n. *Rhodoligotrophaceae*, the *Rhodoligotrophos* family).

The description is as given for *Rhodoligotrophos* [39], which is the type and currently the sole genus of the family. This family can be differentiated from other families based on phylogenetic analyses of core proteome sequences and OGRI calculations (cpAAI and wpAAI).

## Description of *Salaquimonadaceae* fam. nov.

*Salaquimonadaceae* (Sal.a.qui.mo’nad.a’ce.ae. N.L. fem. n. *Salaquimonas*, type genus of the family; -*aceae*, ending to denote a family; N.L. fem. pl. n. *Salaquimonadaceae*, the *Salaquimonas* family).

The description is as given for *Salaquimonas* [40], which is the type and currently the sole genus of the family. This family can be differentiated from other families based on phylogenetic analyses of core proteome sequences and OGRI calculations (cpAAI and wpAAI).

## Emended description of *Bartonellaceae* Gieszczykiewicz 1939 (Approved Lists 1980) emend. Brenner *et al*. 1993

The description is based on descriptions provided previously [3,41,45]. Cells are Gram-negative, aerobic, with variable morphology (rod, ovoid, coccoid, coccobacilli, or ring or disc shaped) and non-spore-forming. Cells can be motile by means of flagella, or non-motile. They are generally catalase and oxidase positive. Predominantly aerobic or facultatively anaerobic heterotrophs, with some species able to utilize carbohydrates while others cannot. The optimum growth temperature ranges between 20 and 37 °C. The predominant respiratory quinone is generally Q-10. Some species are human pathogens. The G+C content as calculated from genome sequences of the type strains of type species of genera within this family is 38.2–65.1 mol%. Genera currently assigned to the family *Bartonellaceae* are listed in Table 2.

## supplementary material

10.1099/ijsem.0.006328 Supplementary Material 1.

10.1099/ijsem.0.006328 Uncited Fig. S1.

10.1099/ijsem.0.006328 Uncited Table S1.

## Abbreviations

AAI: average amino acid identity
BIC: bayesian information criterion
core_138: core genes of the 138 strains
core_143: core genes of the 143 strains
cpAAI: core-proteome average amino acid identity
LPSN: list of prokaryotic names with standing in nomenclature
ML: maximum-likelihood
NCBI: National Center for Biotechnology Information
NSERC: Natural Sciences and Engineering Research Council of Canada
OGRI: overall genomic relatedness indices
perc95_138: genes found in at least 95% of the 138 strains
perc95_143: genes found in at least 95% of the 143 strains
SD: standard deviation
SH-aLRT: Shimodaira–Hasegawa-like approximate likelihood ratio test
wpAAI: whole-proteome average amino acid identity
