# Supplementary material for: Refining the taxonomy of the order Hyphomicrobiales (Rhizobiales) based on whole genome comparisons of over 130 type strains
Source: Int J Syst Evol Microbiol. 2024 Apr 15;74(4):006328. doi: 10.1099/ijsem.0.006328 (PMC11092082; doi:10.1099/ijsem.0.006328)
Supplement: Supplementary Material 1. [file ijsem-74-06328-s001.pdf]

Supplementary Material for
Refining the taxonomy of the order *Hyphomicrobiales* (*Rhizobiales*) based on whole genome comparisons of over 130 type strains

George C. diCenzo, Yuqi Yang, J. Peter W. Young, Nemanja Kuzmanović,

George diCenzo
Email: george.dicenzo@queensu.ca

Nemanja Kuzmanović
Email: kuzmanovic1306@gmail.com; nemanja.kuzmanovic@julius-kuehn.de

This PDF file includes:

Text S1 to S2 (pages 2-3)
Legends for Figures S1 to S12 (pages 4-7)
Legends for Datasets S1 to S17 (page 8)

Other supplementary material for this manuscript is available via FigShare and includes:

Figures S1 to S12
Datasets S1 to S17

Text S1

Preliminary investigation of the perc95_143 gene set (i.e., the 256 marker genes identified in at least 95% of the 143 strains included in our dataset) identified five strains whose publicly available genome lacked >10% of these genes: *Liberibacter crescens* BT-1^{T}, *Methylobrevis pamukkalensis* VKM b-2849^{T}, *Chenggangzhangella methanolivorans* CHL1^{T}, *Nitratireductor aquibiodomus* JCM 21793^{T}, and *Methyloligella halotolerans* VKM B-2706^{T}. Eliminating these five strains from our dataset more than tripled the number of non-recombining core genes from 19 to 59. As our dataset included at least one additional representative from each of the families containing these five strains, we repeated the phylogenetic and OGRI analyses without these strains to obtain more robust results.

The low perc95_143 prevalence in *L. crescens* BT-1^{T} is likely a consequence of its highly reduced genome, although we note that *Bartonella bacilliformis* KC583^{T} has a similar genome size but contained orthologs of all perc95_143 genes. The CheckM completion scores calculated from the proteomes of *L. crescens* BT-1^{T} was 94%, consistent with the low prevalence of the perc95_143 gene set in this strain being a result of its reduced genome rather than a poor quality assembly.

The low perc95_143 prevalence in *M. pamukkalensis* VKM b-2849^{T}, *C. methanolivorans* CHL1^{T}, and *N. aquibiodomus* JCM 21793^{T} is likely a consequence of the high percentage of pseudogenes in these genomes (16% of genes are annotated as pseudogenes). While this high rate of pseudogenes may be accurate, we cannot rule out that it was caused by errors in the genome assemblies. Consistent with the high pseudogene count being a result of sequencing and/or assembly errors, the CheckM completion scores calculated from the proteomes of *M. pamukkalensis* VKM b-2849^{T}, *C. methanolivorans* CHL1^{T}, and *N. aquibiodomus* JCM 21793^{T} were 75%, 69%, and 77%, respectively.

The reason for the low perc95_143 prevalence in *M. halotolerans* VKM B-2706^{T} was less obvious. However, it may reflect a combination of the below average protein-coding gene count (2,872 vs an average of 4,294), the above average pseudogene rate (7.3% versus 2.5%), and the genome assembly being in contigs rather than fully assembled; the CheckM completion scores calculated from the proteome of *M. halotolerans* VKM B-2706^{T} was 88%.

Text S2

As members of the genus *Bartonella* have highly reduced genomes, we chose to perform a secondary analysis to be more confident that the genus *Bartonella* falls within the monophyletic group that includes the families *Phyllobacteriaceae*, *Notoacmeibacteraceae*, and *Brucellaceae*. To this end, we repeated our phylogenetic analyses using a taxonomically restricted subset of the *Hyphomicrobiales* dataset, including the families *Phyllobacteriaceae*, *Notoacmeibacteraceae*, *Brucellaceae*, *Bartonellaceae*, *Ahrensiaceae*, *Rhizobiaceae*, and *Aurantimonadaceae*. In addition, we supplemented our dataset with four additional *Bartonella* species type strains (*Bartonella apis* PEB0122^T, *Bartonella birtlesii* IBS 325^T, *Bartonella henselae* ATCC 49882^T, and *Bartonella krasnovii* OE 1-1^T) that represent the full taxonomic breadth of the genus [1], as well as *Flavimaribacter sediminis* WL0058^T proposed to belong to the family *Rhizobiaceae*. This yielded a final dataset of 58 strains (Dataset S3).

GET_HOMOLOGUES and GET_PHYLOMARKERS were used to identify non-recombining single-copy marker genes present in all 58 genomes. This led to the identification of 120 core genes (termed core_58). In addition, GET_HOMOLOGUES was used to identify 454 single-copy marker genes present in at least 95% of the strains (termed perc95_58). Repeating these analyses with a reduced set of 56 genomes lacking *L. crescens* BT-1^T and *N. aquibiodomus* JCM 21793^T (see Text S1) led to the identification of 178 core genes present in all strains (termed core_56), and 497 genes present in at least 95% of the genomes (termed perc95_56). As described in the Methods section of the main manuscript for the *Hyphomicrobiales* dataset, the proteins encoded by the four gene sets were used to construct maximum likelihood phylogenies using IQ-TREE with the best scoring model (core_58: LG+F+R6; perc95_58: LG+F+R8; core_56: LG+F+R5; perc95_56: LG+F+R9).

The five species type strains of the genus *Bartonella* formed a monophyletic group in all four of the resulting phylogenies, as expected (Figures S8-S11). Importantly, all four phylogenies placed the genus *Bartonella* within the monophyletic group that also included the families *Phyllobacteriaceae*, *Notoacmeibacteraceae*, and *Brucellaceae* (Figures S8-S11) although the exact position differed compared to the position of *B. bacilliformis* KC583^T in the full *Hyphomicrobiales* phylogenies (Figures 2, and S1-S3). Collectively, these results are consistent with the proposal to unify the families *Bartonellaceae*, *Brucellaceae*, *Notoacmeibacteraceae*, and *Phyllobacteriaceae* under the name *Bartonellaceae*. In addition, the four supplemental phylogenies supported the placement of the recently proposed genus *Flavimaribacter* within the family *Rhizobiaceae* (Figures S8-S11).

1. **Gutiérrez R, Shalit T, Markus B, Yuan C, Nachum-Biala Y, *et al.*** *Bartonella kosoyi* sp. nov. and *Bartonella krasnovii* sp. nov., two novel species closely related to the zoonotic *Bartonella elizabethae*, isolated from black rats and wild desert rodent-fleas. *Int J of Syst Evol Microbiol* 2020;70:1656–1665.

SUPPLEMENTARY FIGURE LEGENDS

Figure S1. Phylogenetic and core-proteome AAI (cpAA) analyses of the order *Hyphomicrobiales*. On the left, a maximum likelihood phylogeny of 138 *Hyphomicrobiales* type strains is shown, built using the concatenated protein alignments encoded by the perc95_143 gene set (256 genes present in at least 95% of the strains). The phylogeny was rooted using five *Caulobacterales* type strains as the outgroup. The numbers on the nodes indicate the ultra-fast jackknife values using a 40% resampling rate (top numbers) and the SH-aLRT support values (bottom numbers), both calculated from 1000 replicates. Only values below 100 are shown. The scale bar represents the average number of amino acid substitutions per site. To the right of the phylogeny is the current family assignments of each of the 138 *Hyphomicrobiales* type strains, followed to the right by the proposed family assignments of each strain. On the righthand side, a matrix is provided showing the cpAAI values between each pair of strains calculated using the proteins encoded by the core_143 gene set (19 genes present in 100% of the strains). Values less than 78% are in white while all values greater than 88% are the same shade of blue. Black boxes indicate the proposed families.

Figure S2. Phylogenetic analysis of the order *Hyphomicrobiales*. On the left, a maximum likelihood phylogeny of 138 *Hyphomicrobiales* type strains is shown, built using the concatenated protein alignments encoded by the core_143 gene set (19 genes present in 100% of the strains). The phylogeny was rooted using five *Caulobacterales* type strains as the outgroup. The numbers on the nodes indicate the ultra-fast jackknife values using a 40% resampling rate (top numbers) and the SH-aLRT support values (bottom numbers), both calculated from 1000 replicates. Only values below 100 are shown. The scale bar represents the average number of amino acid substitutions per site. To the right of the phylogeny is the current family assignments of each of the 138 *Hyphomicrobiales* type strains, followed to the right by the proposed family assignments of each strain.

Figure S3. Phylogenetic analysis of the order *Hyphomicrobiales*. On the left, a maximum likelihood phylogeny of 133 *Hyphomicrobiales* type strains is shown, built using the concatenated protein alignments encoded by the core_138 gene set (59 genes present in 100% of the strains). The phylogeny was rooted using five *Caulobacterales* type strains as the outgroup. The numbers on the nodes indicate the ultra-fast jackknife values using a 40% resampling rate (top numbers) and the SH-aLRT support values (bottom numbers), both calculated from 1000 replicates. Only values below 100 are shown. The scale bar represents the average number of amino acid substitutions per site. To the right of the phylogeny is the current family assignments of each of the 133 *Hyphomicrobiales* type strains, followed to the right by the proposed family assignments of each strain.

Figure S4. Phylogenetic and whole-proteome AAI (wpAA) analyses of the order *Hyphomicrobiales*. On the left, a maximum likelihood phylogeny of 138 *Hyphomicrobiales* type strains is shown, built using the concatenated protein alignments encoded by the perc95_143 gene set (256 genes present in at least 95% of the strains). The phylogeny was rooted using five *Caulobacterales* type strains as the outgroup. The numbers on the nodes indicate the ultra-fast jackknife values using a 40% resampling rate (top numbers) and the SH-aLRT support values (bottom numbers), both calculated from 1000 replicates. Only values below 100 are shown. The scale bar represents the average number of amino acid substitutions per site. To the right of the phylogeny is the current family assignments of each of the 138 *Hyphomicrobiales* type strains, followed to the right by the proposed family assignments of each strain. On the righthand side, a matrix is provided showing the wpAAI values between each pair of strains calculated using EzAAI. Values less than 63% are in white while all values greater than 73% are the same shade of blue. Black boxes indicate the proposed families.

Figure S5. Distribution of core-proteome AAI (cpAAI) comparisons of the order *Hyphomicrobiales*. Pairwise cpAAI values were calculated based on 19 nonrecombinant loci from the core genome of 138 members of the order *Hyphomicrobiales* and five members of the order *Caulobacterales*. Results are summarized as histograms with a bin width of 1%. The cpAAI values calculated between two strains belonging to different orders (yellow), different families but same order (blue), or the same family (red) are summarized separately. Dashed vertical lines represent the mean value of each distribution. In all plots, cpAAI values where both strains belong to the order *Caulobacterales* were excluded. (**A**) The distribution of all pairwise cpAAI values with the classification (i.e., between order, between family, or within family) based on existing taxonomic assignments. (**B**) The distribution of all pairwise cpAAI values except for those including at least one strain from the family *Rhizobiaceae*, with the classification based on existing taxonomic assignments. (**C**) The distribution of all pairwise cpAAI values with the classification based on the proposed taxonomic assignments. (**D**) The distribution of all pairwise cpAAI values except for those including at least one strain from the family *Rhizobiaceae*, with the classification based on the proposed taxonomic assignments.

Figure S6. Distribution of whole-proteome AAI (wpAAI) comparisons of the order *Hyphomicrobiales*. Pairwise wpAAI values were calculated between 138 members of the order *Hyphomicrobiales* and five members of the order *Caulobacterales*. Results are summarized as histograms with a bin width of 1%. The wpAAI values calculated between two strains belonging to different orders (yellow), different families but same order (blue), or the same family (red) are summarized separately. Dashed vertical lines represent the mean value of each distribution. In all plots, wpAAI values where both strains belong to the order *Caulobacterales* were excluded. (**A**) The distribution of all pairwise wpAAI values with the classification (i.e., between order, between family, or within family) based on existing taxonomic assignments. (**B**) The distribution of all pairwise wpAAI values except for those including at least one strain from the family *Rhizobiaceae*, with the classification based on existing taxonomic assignments. (**C**) The distribution of all pairwise wpAAI values with the classification based on the proposed taxonomic assignments. (**D**) The distribution of all pairwise wpAAI values except for those including at least one strain from the family *Rhizobiaceae*, with the classification based on the proposed taxonomic assignments.

Figure S7. 16S rRNA gene phylogenetic analysis of the order *Hyphomicrobiales*. On the left, a maximum likelihood phylogeny of 162 *Hyphomicrobiales* type strains is shown, built using a trimmed, Clustal Omega alignment of the 16S rRNA genes. The phylogeny was rooted using five *Caulobacterales* type strains as the outgroup. In cases where a genome sequence contained more than one 16S rRNA gene sequence, all unique 16S rRNA gene sequences were included. The numbers on the nodes indicate the ultra-fast bootstrap support values (top numbers) and the SH-aLRT support values (bottom numbers), both calculated from 1000 replicates. The scale bar represents the average number of nucleotide substitutions per site. To the right of the phylogeny is the current family assignments of each of the 162 *Hyphomicrobiales* type strains, followed to the right by the proposed family assignments of each strain.

Figure S8. Phylogenetic analysis of the family *Bartonellaceae* and related families. On the left, an unrooted maximum likelihood phylogeny of 58 *Hyphomicrobiales* type strains is shown, built using the concatenated protein alignments encoded by the core_58 gene set (120 genes present in 100% of the included strains). The numbers on the nodes indicate the ultra-fast jackknife values using a 40% resampling rate (top numbers) and the SH-aLRT support values (bottom numbers), both calculated from 1000 replicates. Only values below 100 are shown. The scale bar represents the average number of amino acid substitutions per site. To the right of the phylogeny is the current family assignments of

each of the 58 strains, followed to the right by the proposed family assignments of each strain.

Figure S9. Phylogenetic analysis of the family *Bartonellaceae* and related families. On the left, an unrooted maximum likelihood phylogeny of 58 *Hyphomicrobiales* type strains is shown, built using the concatenated protein alignments encoded by the perc95_58 gene set (454 genes present in at least 95% of the included strains). The numbers on the nodes indicate the ultra-fast jackknife values using a 40% resampling rate (top numbers) and the SH-aLRT support values (bottom numbers), both calculated from 1000 replicates. Only values below 100 are shown. The scale bar represents the average number of amino acid substitutions per site. To the right of the phylogeny is the current family assignments of each of the 58 strains, followed to the right by the proposed family assignments of each strain.

Figure S10. Phylogenetic analysis of the family *Bartonellaceae* and related families. On the left, an unrooted maximum likelihood phylogeny of 56 *Hyphomicrobiales* type strains is shown, built using the concatenated protein alignments encoded by the core_56 gene set (178 genes present in 100% of the included strains). The numbers on the nodes indicate the ultra-fast jackknife values using a 40% resampling rate (top numbers) and the SH-aLRT support values (bottom numbers), both calculated from 1000 replicates. Only values below 100 are shown. The scale bar represents the average number of amino acid substitutions per site. To the right of the phylogeny is the current family assignments of each of the 58 strains, followed to the right by the proposed family assignments of each strain.

Figure S11. Phylogenetic analysis of the family *Bartonellaceae* and related families. On the left, an unrooted maximum likelihood phylogeny of 56 *Hyphomicrobiales* type strains is shown, built using the concatenated protein alignments encoded by the perc95_56 gene set (497 genes present in at least 95% of the included strains). The numbers on the nodes indicate the ultra-fast jackknife values using a 40% resampling rate (top numbers) and the SH-aLRT support values (bottom numbers), both calculated from 1000 replicates. Only values below 100 are shown. The scale bar represents the average number of amino acid substitutions per site. To the right of the phylogeny is the current family assignments of each of the 58 strains, followed to the right by the proposed family assignments of each strain.

Figure S12. Validation of the proposed taxonomic framework. Our pipeline for assigning genera to families in the order *Hyphomicrobiales* was run on five genomes not included in our original analyses. Shown is the resulting maximum likelihood phylogeny, including 133 *Hyphomicrobiales* type strains from our original dataset (black font) and the five new genomes (red font). The phylogeny was inferred from the concatenated protein alignments encoded by the perc95_138 gene set, and five *Caulobacterales* type strains were included as the outgroup to root the phylogeny. The numbers on the nodes indicate the ultra-fast jackknife values using a 40% resampling rate (top numbers) and the SH-aLRT support values (bottom numbers), both calculated from 1000 replicates. Only values below 100 are shown. The scale bar represents the average number of amino acid substitutions per site. The numbers to the right of the phylogeny show the pairwise cpAAI values between a new genome and a neighbouring taxon; in all cases these numbers are greater than 75% indicating the strains belong to the same family. To the right of this are the current family assignments of each of the 138 *Hyphomicrobiales* type strains, followed to the right by the proposed family assignments of each strain.

DATASET LEGENDS

Dataset S1. Metadata for the 138 *Hyphomicrobiales* strains used in the primary analyses reported in this study, including ftp links to download the genomes.

Dataset S2. Metadata for the 5 *Hyphomicrobiales* strains used in this study, including ftp links to download the genomes.

Dataset S3. Metadata for the 53 *Hyphomicrobiales* strains used in the secondary analyses of the *Bartonellaceae* and related families, including ftp links to download the genomes.

Dataset S4. List of organisms for which the 16S rRNA gene sequences were downloaded from LPSN rather than extracted from the whole genome sequences, including the GenBank accession number of each sequence.

Dataset S5. A matrix of all pairwise cpAAI values calculated from the proteins encoded by the core_143 gene set.

Dataset S6. A matrix of all pairwise cpAAI values calculated from the proteins encoded by the core_138 gene set.

Dataset S7. A matrix of all pairwise wpAAI values calculated using EzAAI.

Dataset S8. The phylogeny of Figure 1 provided in Newick format.

Dataset S9. The phylogeny of Figure S1 and S4 provided in Newick format.

Dataset S10. The phylogeny of Figure S2 provided in Newick format.

Dataset S11. The phylogeny of Figure S3 provided in Newick format.

Dataset S12. The phylogeny of Figure S7 provided in Newick format.

Dataset S13. The phylogeny of Figure S8 provided in Newick format.

Dataset S14. The phylogeny of Figure S9 provided in Newick format.

Dataset S15. The phylogeny of Figure S10 provided in Newick format.

Dataset S16. The phylogeny of Figure S11 provided in Newick format.

Dataset S17. The phylogeny of Figure S12 provided in Newick format.
