# Supplementary material for: Refining the taxonomy of the order Hyphomicrobiales (Rhizobiales) based on whole genome comparisons of over 130 type strains
Source: Int J Syst Evol Microbiol. 2024 Apr 15;74(4):006328. doi: 10.1099/ijsem.0.006328 (PMC11092082; doi:10.1099/ijsem.0.006328)
Supplement: Uncited Fig. S1. [file ijsem-74-06328-s002.pdf]

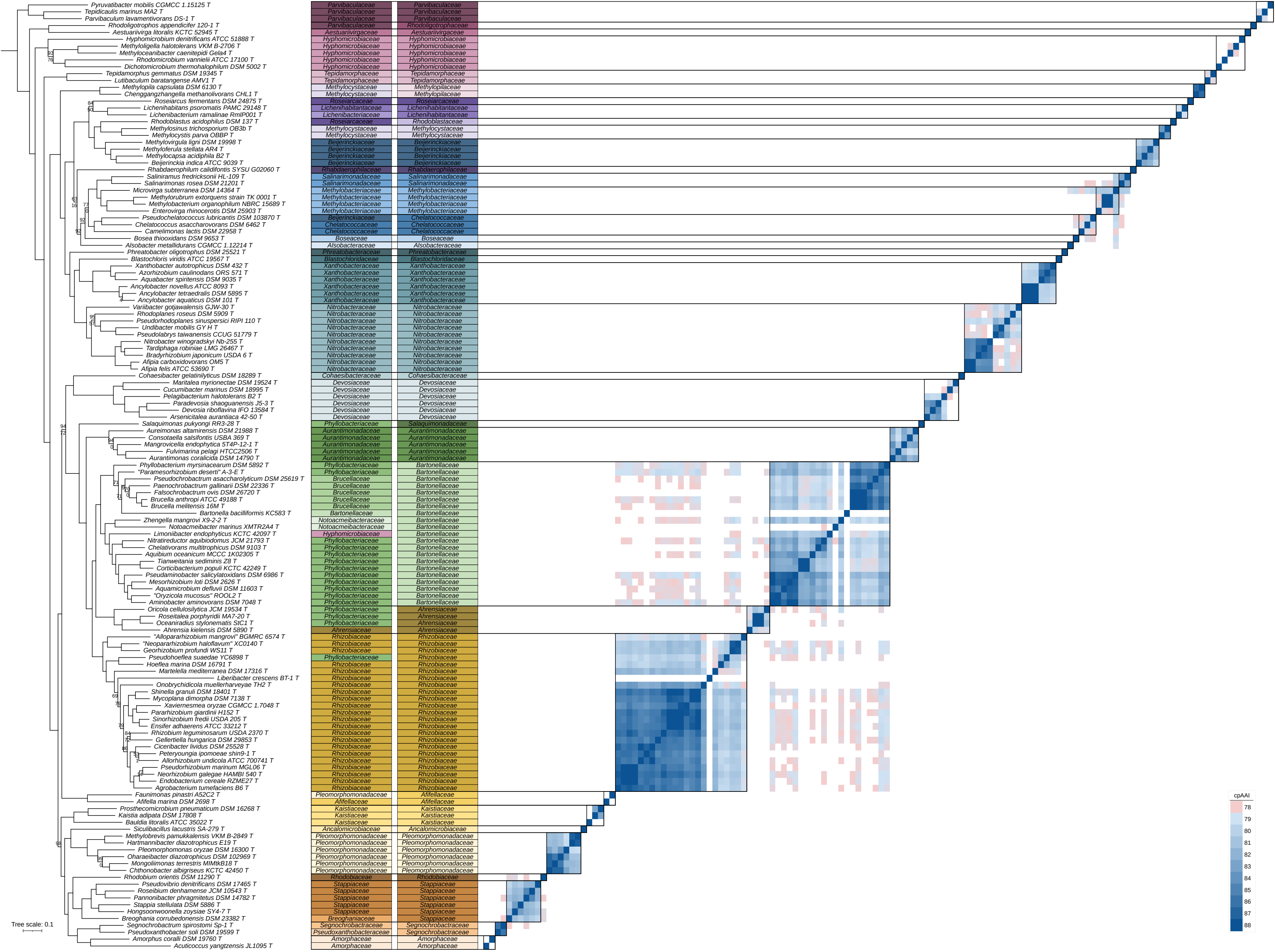

Figure S1. Phylogenetic and core-proteome AAI (cpAA) analyses of the order *Hyphomicrobiales*. On the left, a maximum likelihood phylogeny of 138 *Hyphomicrobiales* type strains is shown, built using the concatenated protein alignments encoded by the perc95_143 gene set (256 genes present in at least 95% of the strains). The phylogeny was rooted using five *Caulobacterales* type strains as the outgroup. The numbers on the nodes indicate the ultra-fast jackknife values using a 40% resampling rate (top numbers) and the SH-aLRT support values (bottom numbers), both calculated from 1000 replicates. Only values below 100 are shown. The scale bar represents the average number of amino acid substitutions per site. To the right of the phylogeny is the current family assignments of each of the 138 *Hyphomicrobiales* type strains, followed to the right by the proposed family assignments of each strain. On the righthand side, a matrix is provided showing the cpAAI values between each pair of strains calculated using the proteins encoded by the core_143 gene set (19 genes present in 100% of the strains). Values less than 78% are in white while all values greater than 88% are the same shade of blue. Black boxes indicate the proposed families.

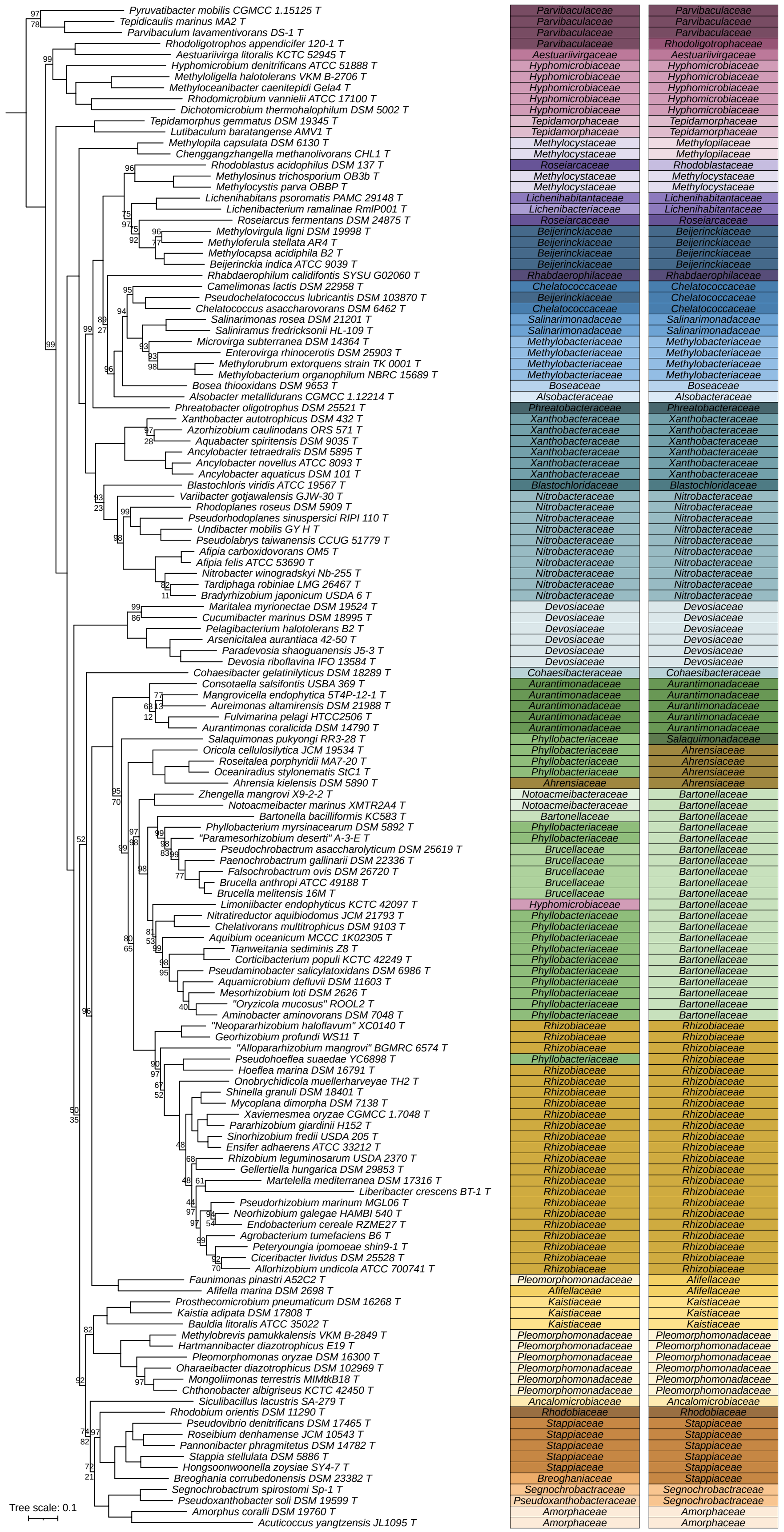

Figure S2. Phylogenetic analysis of the order *Hyphomicrobiales*. On the left, a maximum likelihood phylogeny of 138 *Hyphomicrobiales* type strains is shown, built using the concatenated protein alignments encoded by the core_143 gene set (19 genes present in 100% of the strains). The phylogeny was rooted using five *Caulobacterales* type strains as the outgroup. The numbers on the nodes indicate the ultra-fast jackknife values using a 40% resampling rate (top numbers) and the SH-aLRT support values (bottom numbers), both calculated from 1000 replicates. Only values below 100 are shown. The scale bar represents the average number of amino acid substitutions per site. To the right of the phylogeny is the current family assignments of each of the 138 *Hyphomicrobiales* type strains, followed to the right by the proposed family assignments of each strain.

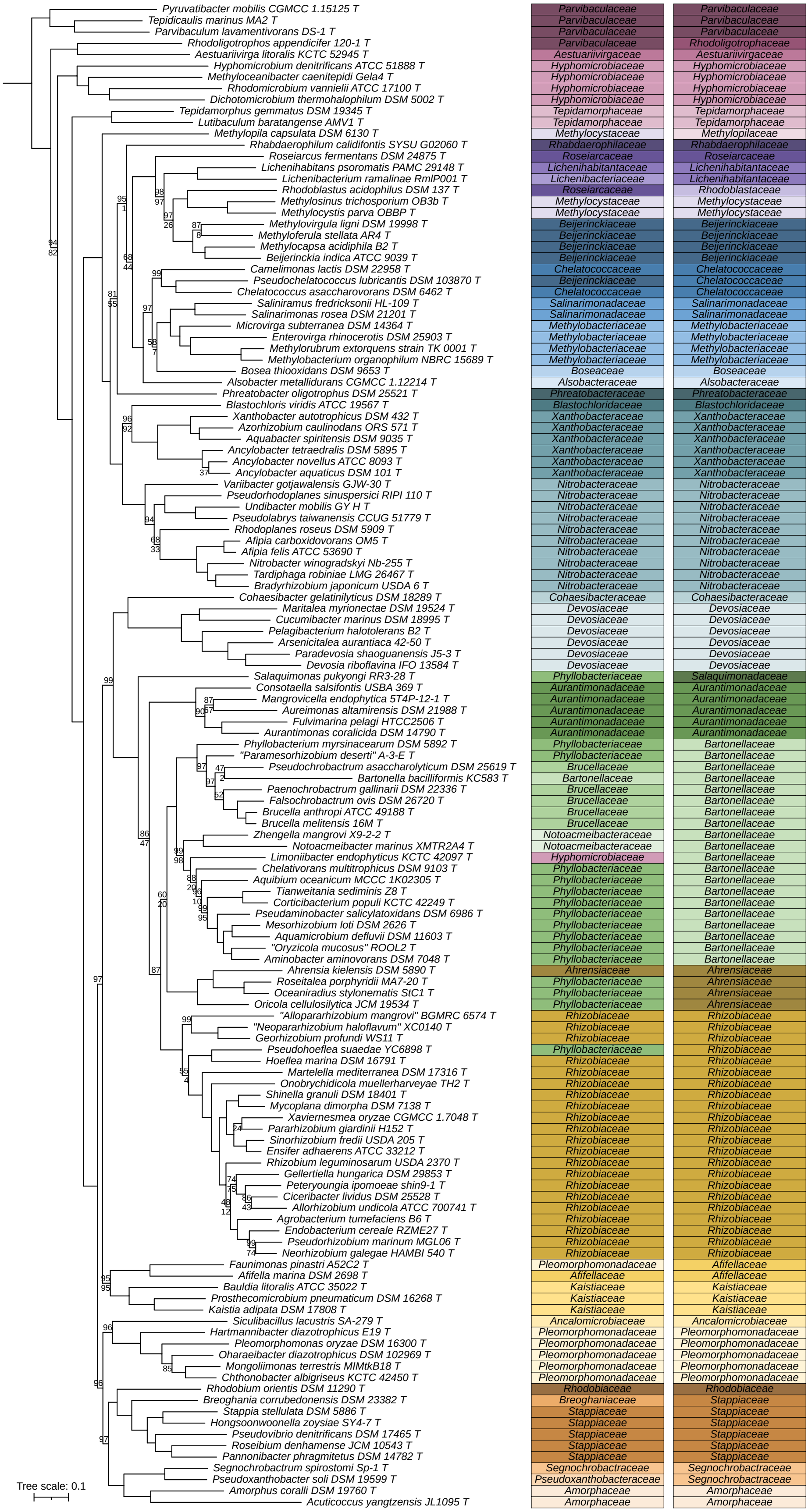

Figure S3. Phylogenetic analysis of the order *Hyphomicrobiales*. On the left, a maximum likelihood phylogeny of 133 *Hyphomicrobiales* type strains is shown, built using the concatenated protein alignments encoded by the core_138 gene set (59 genes present in 100% of the strains). The phylogeny was rooted using five *Caulobacterales* type strains as the outgroup. The numbers on the nodes indicate the ultra-fast jackknife values using a 40% resampling rate (top numbers) and the SH-aLRT support values (bottom numbers), both calculated from 1000 replicates. Only values below 100 are shown. The scale bar represents the average number of amino acid substitutions per site. To the right of the phylogeny is the current family assignments of each of the 133 *Hyphomicrobiales* type strains, followed to the right by the proposed family assignments of each strain.

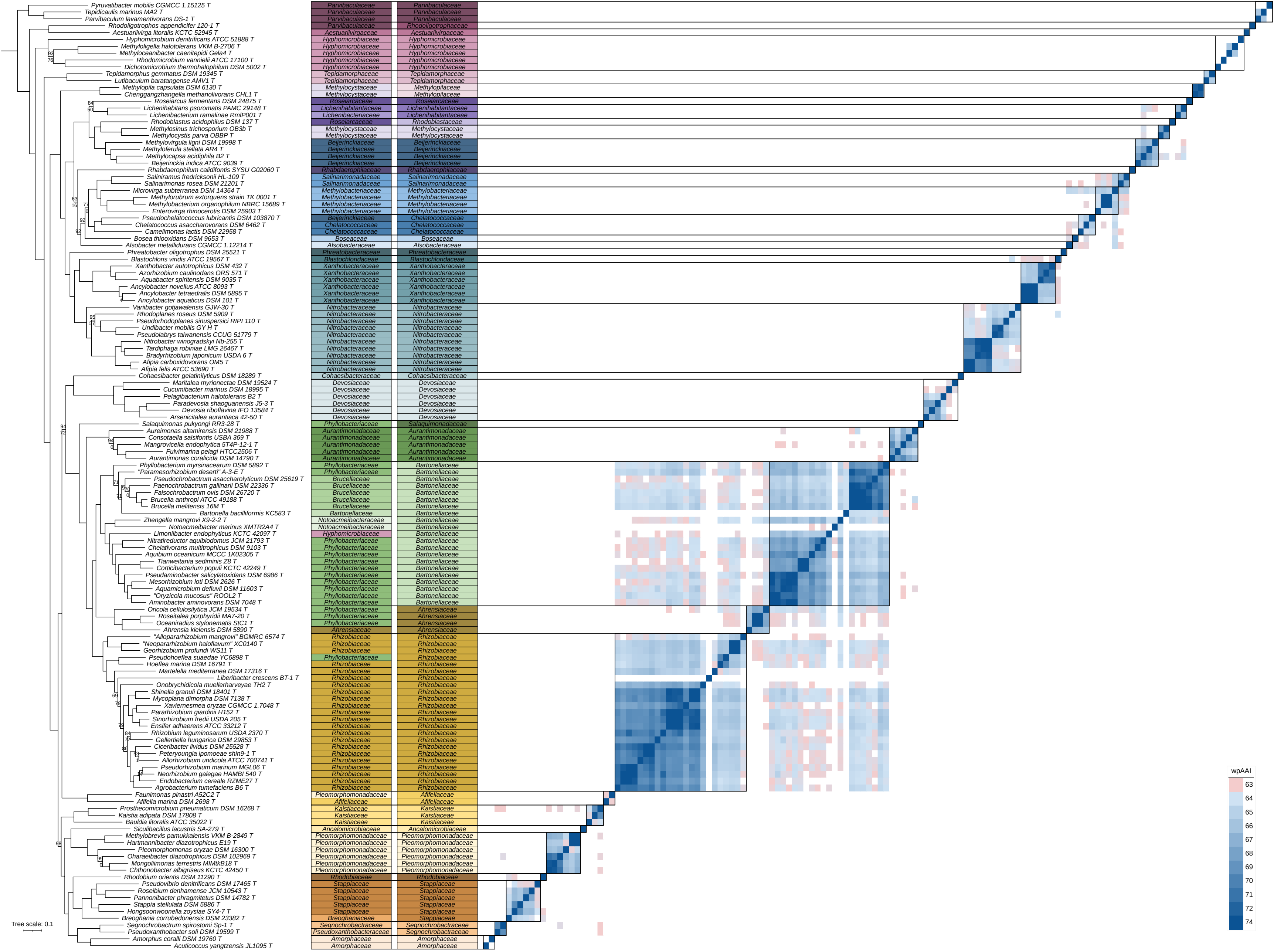

Figure S4. Phylogenetic and whole-proteome AAI (wpAA) analyses of the order *Hyphomicrobiales*. On the left, a maximum likelihood phylogeny of 138 *Hyphomicrobiales* type strains is shown, built using the concatenated protein alignments encoded by the perc95_143 gene set (256 genes present in at least 95% of the strains). The phylogeny was rooted using five *Caulobacterales* type strains as the outgroup. The numbers on the nodes indicate the ultra-fast jackknife values using a 40% resampling rate (top numbers) and the SH-aLRT support values (bottom numbers), both calculated from 1000 replicates. Only values below 100 are shown. The scale bar represents the average number of amino acid substitutions per site. To the right of the phylogeny is the current family assignments of each of the 138 *Hyphomicrobiales* type strains, followed to the right by the proposed family assignments of each strain. On the righthand side, a matrix is provided showing the wpAAI values between each pair of strains calculated using EzAAI. Values less than 63% are in white while all values greater than 73% are the same shade of blue. Black boxes indicate the proposed families.

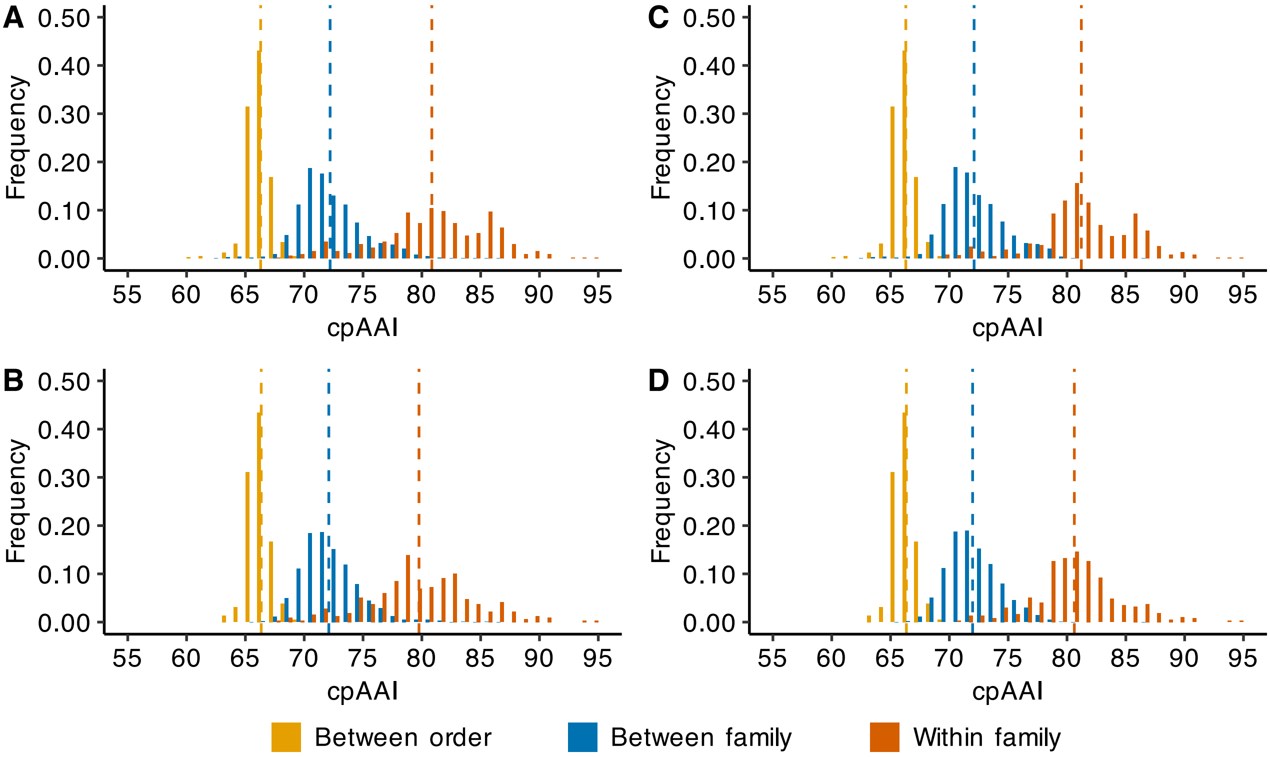

Figure S5. Distribution of core-proteome AAI (cpAAI) comparisons of the order *Hyphomicrobiales*. Pairwise cpAAI values were calculated based on 19 nonrecombinant loci from the core genome of 138 members of the order *Hyphomicrobiales* and five members of the order *Caulobacterales*. Results are summarized as histograms with a bin width of 1%. The cpAAI values calculated between two strains belonging to different orders (yellow), different families but same order (blue), or the same family (red) are summarized separately. Dashed vertical lines represent the mean value of each distribution. In all plots, cpAAI values where both strains belong to the order *Caulobacterales* were excluded. (**A**) The distribution of all pairwise cpAAI values with the classification (i.e., between order, between family, or within family) based on existing taxonomic assignments. (**B**) The distribution of all pairwise cpAAI values except for those including at least one strain from the family *Rhizobiaceae*, with the classification based on existing taxonomic assignments. (**C**) The distribution of all pairwise cpAAI values with the classification based on the proposed taxonomic assignments. (**D**) The distribution of all pairwise cpAAI values except for those including at least one strain from the family *Rhizobiaceae*, with the classification based on the proposed taxonomic assignments.

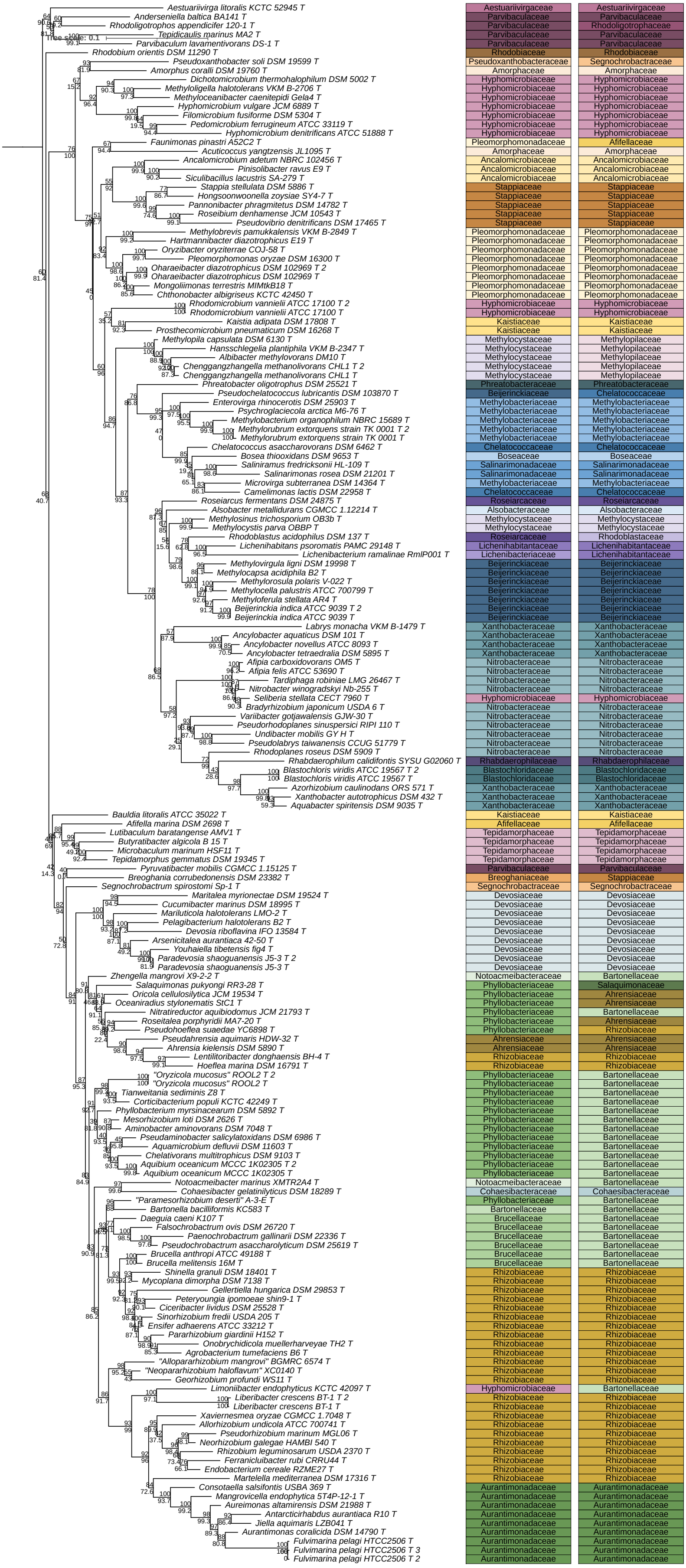

Figure S7. 16S rRNA gene phylogenetic analysis of the order *Hyphomicrobiales*. On the left, a maximum likelihood phylogeny of 162 *Hyphomicrobiales* type strains is shown, built using a trimmed, Clustal Omega alignment of the 16S rRNA genes. The phylogeny was rooted using five *Caulobacterales* type strains as the outgroup. In cases where a genome sequence contained more than one 16S rRNA gene sequence, all unique 16S rRNA gene sequences were included. The numbers on the nodes indicate the ultra-fast bootstrap support values (top numbers) and the SH-aLRT support values (bottom numbers), both calculated from 1000 replicates. The scale bar represents the average number of nucleotide substitutions per site. To the right of the phylogeny is the current family assignments of each of the 162 *Hyphomicrobiales* type strains, followed to the right by the proposed family assignments of each strain.

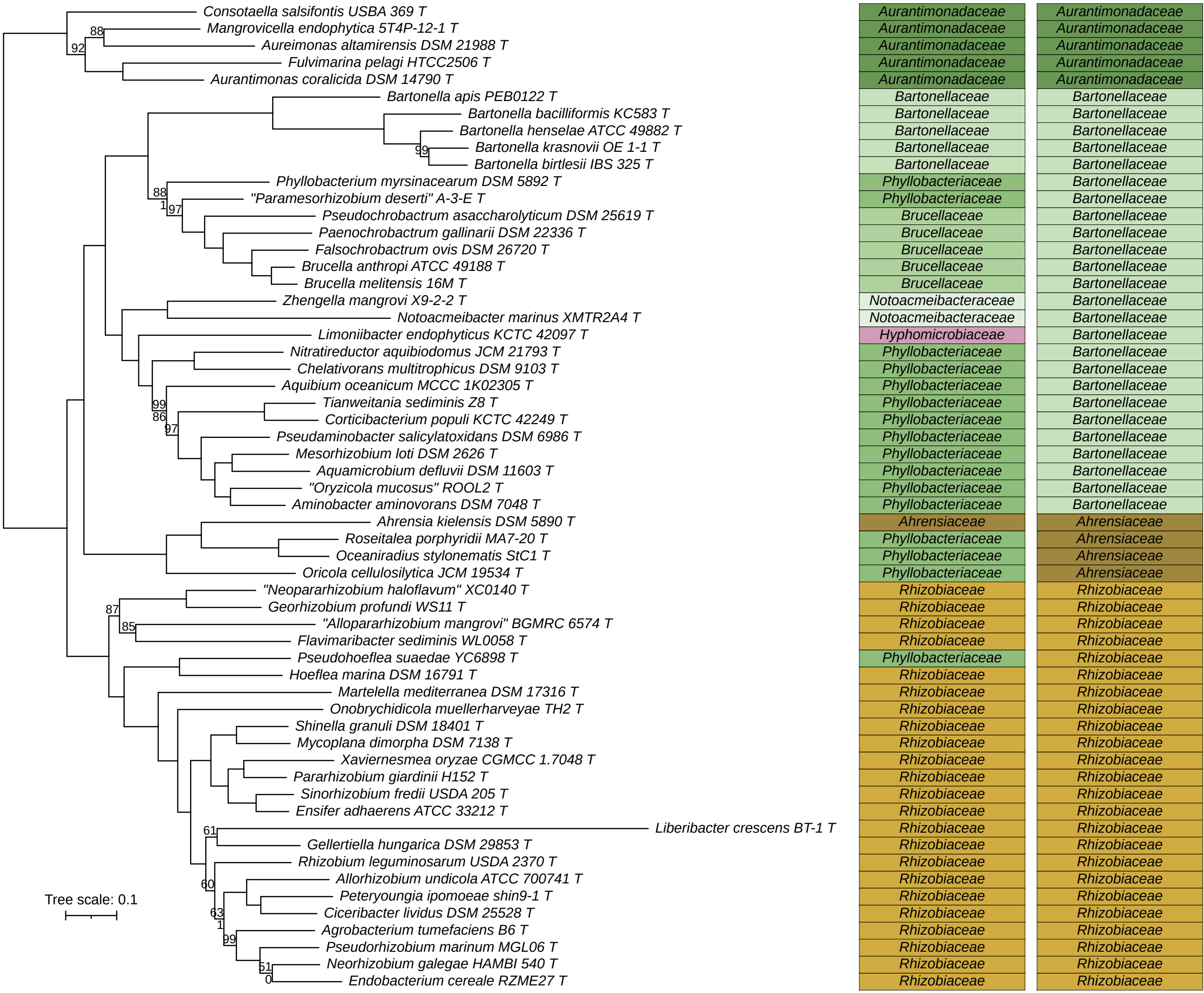

Figure S8. Phylogenetic analysis of the family *Bartonellaceae* and related families. On the left, an unrooted maximum likelihood phylogeny of 58 *Hyphomicrobiales* type strains is shown, built using the concatenated protein alignments encoded by the core_58 gene set (120 genes present in 100% of the included strains). The numbers on the nodes indicate the ultra-fast jackknife values using a 40% resampling rate (top numbers) and the SH-aLRT support values (bottom numbers), both calculated from 1000 replicates. Only values below 100 are shown. The scale bar represents the average number of amino acid substitutions per site. To the right of the phylogeny is the current family assignments of each of the 58 strains, followed to the right by the proposed family assignments of each strain.

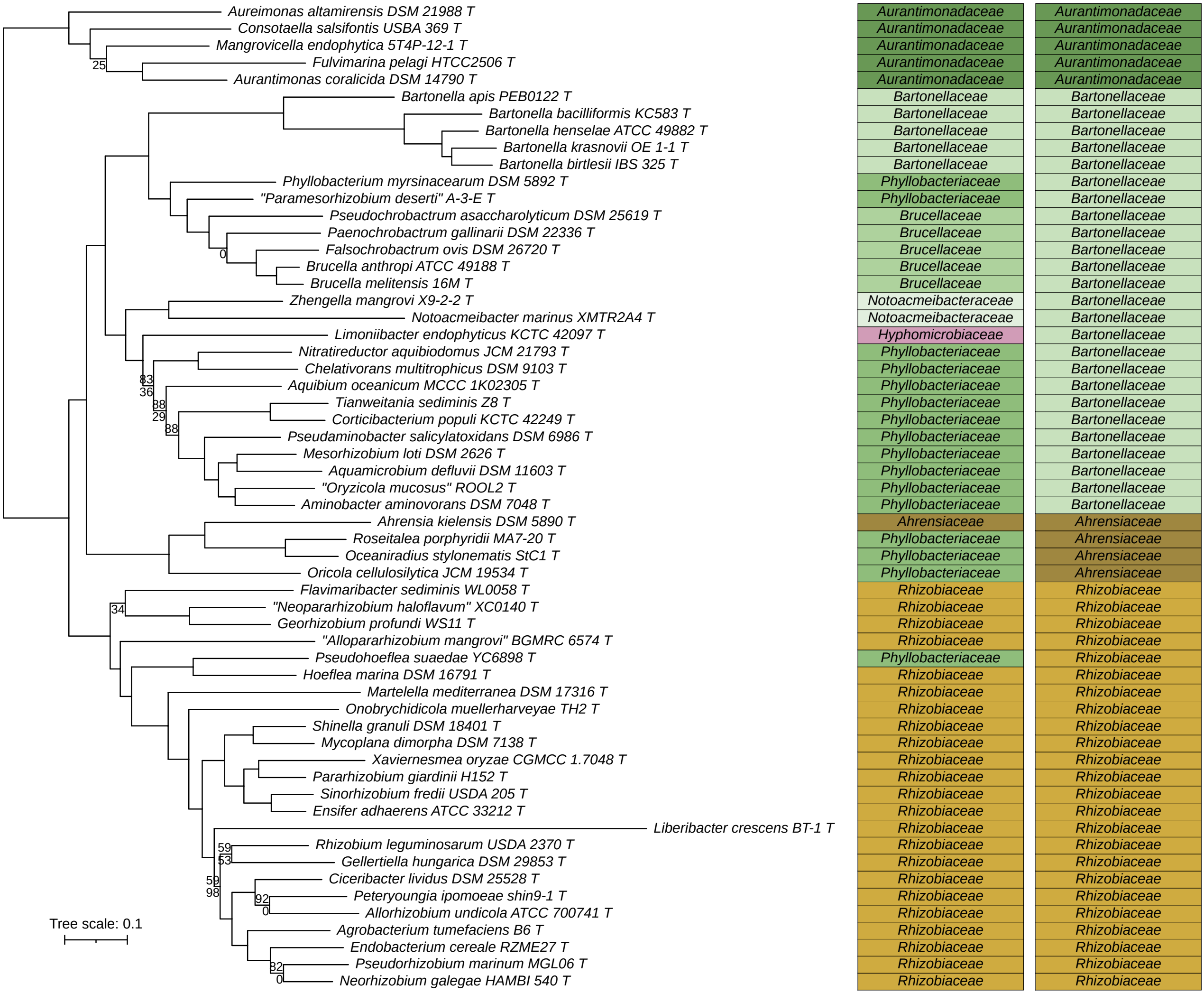

Figure S9. Phylogenetic analysis of the family *Bartonellaceae* and related families. On the left, an unrooted maximum likelihood phylogeny of 58 *Hyphomicrobiales* type strains is shown, built using the concatenated protein alignments encoded by the perc95_58 gene set (454 genes present in at least 95% of the included strains). The numbers on the nodes indicate the ultra-fast jackknife values using a 40% resampling rate (top numbers) and the SH-aLRT support values (bottom numbers), both calculated from 1000 replicates. Only values below 100 are shown. The scale bar represents the average number of amino acid substitutions per site. To the right of the phylogeny is the current family assignments of each of the 58 strains, followed to the right by the proposed family assignments of each strain.

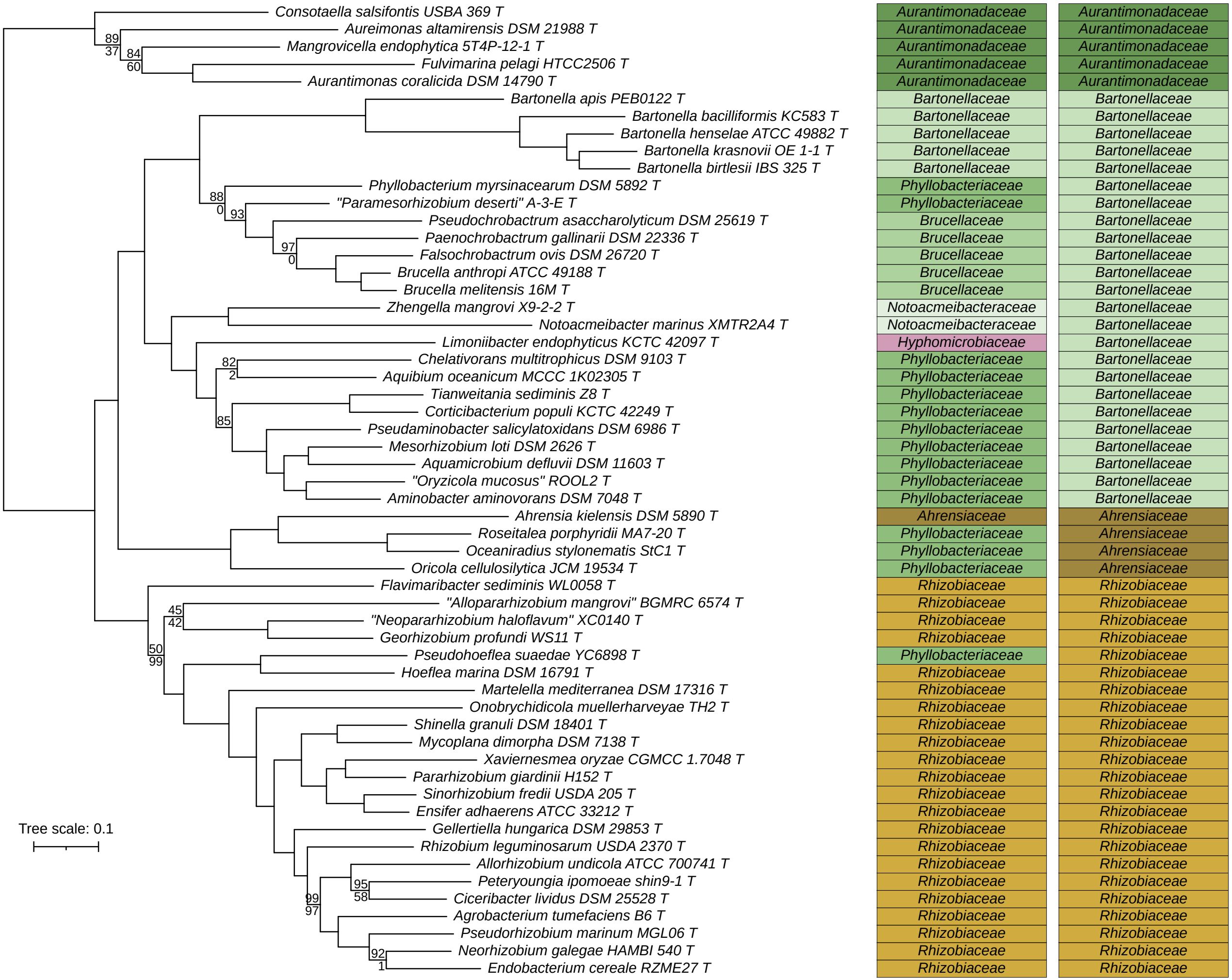

Figure S10. Phylogenetic analysis of the family *Bartonellaceae* and related families. On the left, an unrooted maximum likelihood phylogeny of 56 *Hyphomicrobiales* type strains is shown, built using the concatenated protein alignments encoded by the core_56 gene set (178 genes present in 100% of the included strains). The numbers on the nodes indicate the ultra-fast jackknife values using a 40% resampling rate (top numbers) and the SH-aLRT support values (bottom numbers), both calculated from 1000 replicates. Only values below 100 are shown. The scale bar represents the average number of amino acid substitutions per site. To the right of the phylogeny is the current family assignments of each of the 58 strains, followed to the right by the proposed family assignments of each strain.

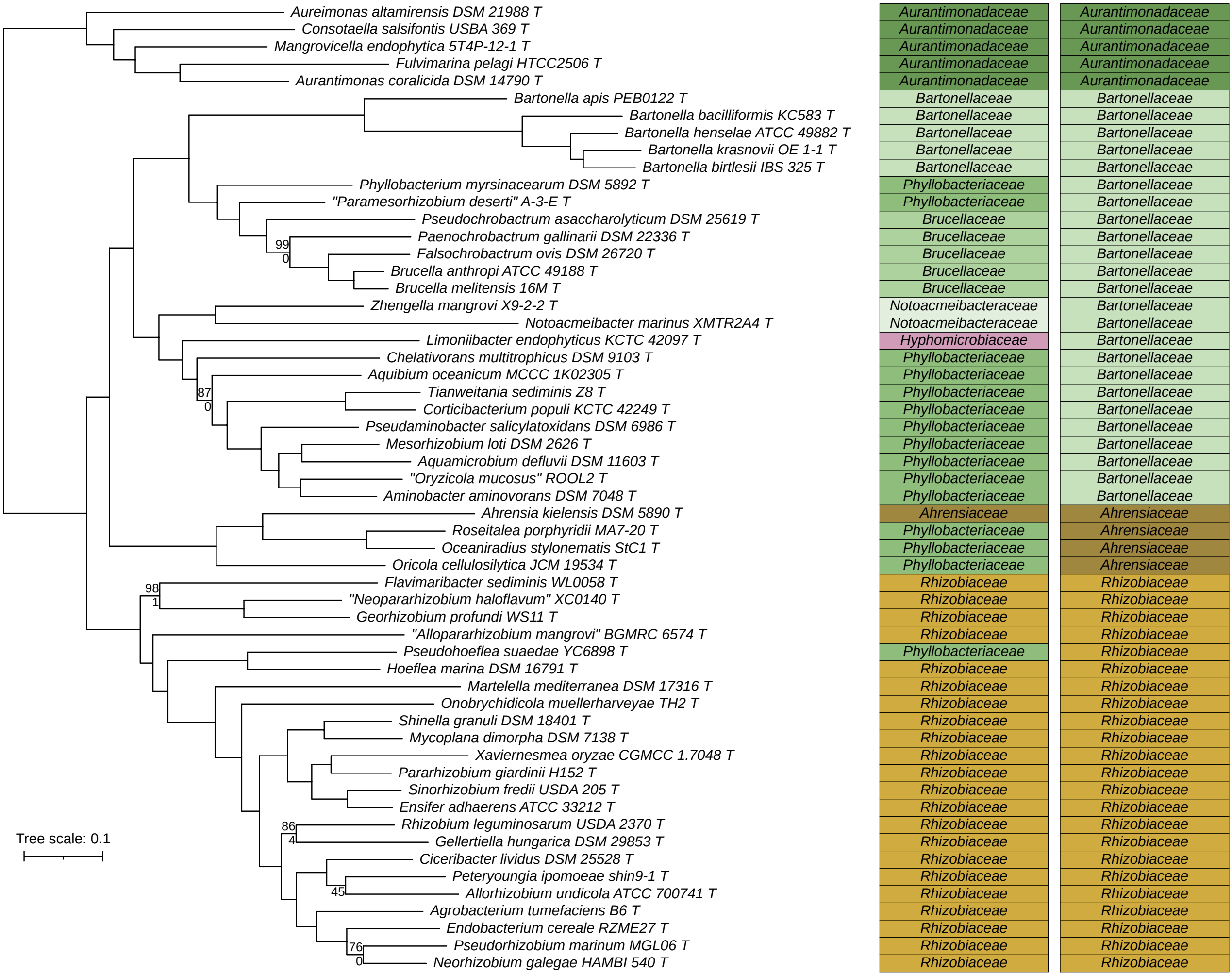

Figure S11. Phylogenetic analysis of the family *Bartonellaceae* and related families. On the left, an unrooted maximum likelihood phylogeny of 56 *Hyphomicrobiales* type strains is shown, built using the concatenated protein alignments encoded by the perc95_56 gene set (497 genes present in at least 95% of the included strains). The numbers on the nodes indicate the ultra-fast jackknife values using a 40% resampling rate (top numbers) and the SH-aLRT support values (bottom numbers), both calculated from 1000 replicates. Only values below 100 are shown. The scale bar represents the average number of amino acid substitutions per site. To the right of the phylogeny is the current family assignments of each of the 58 strains, followed to the right by the proposed family assignments of each strain.

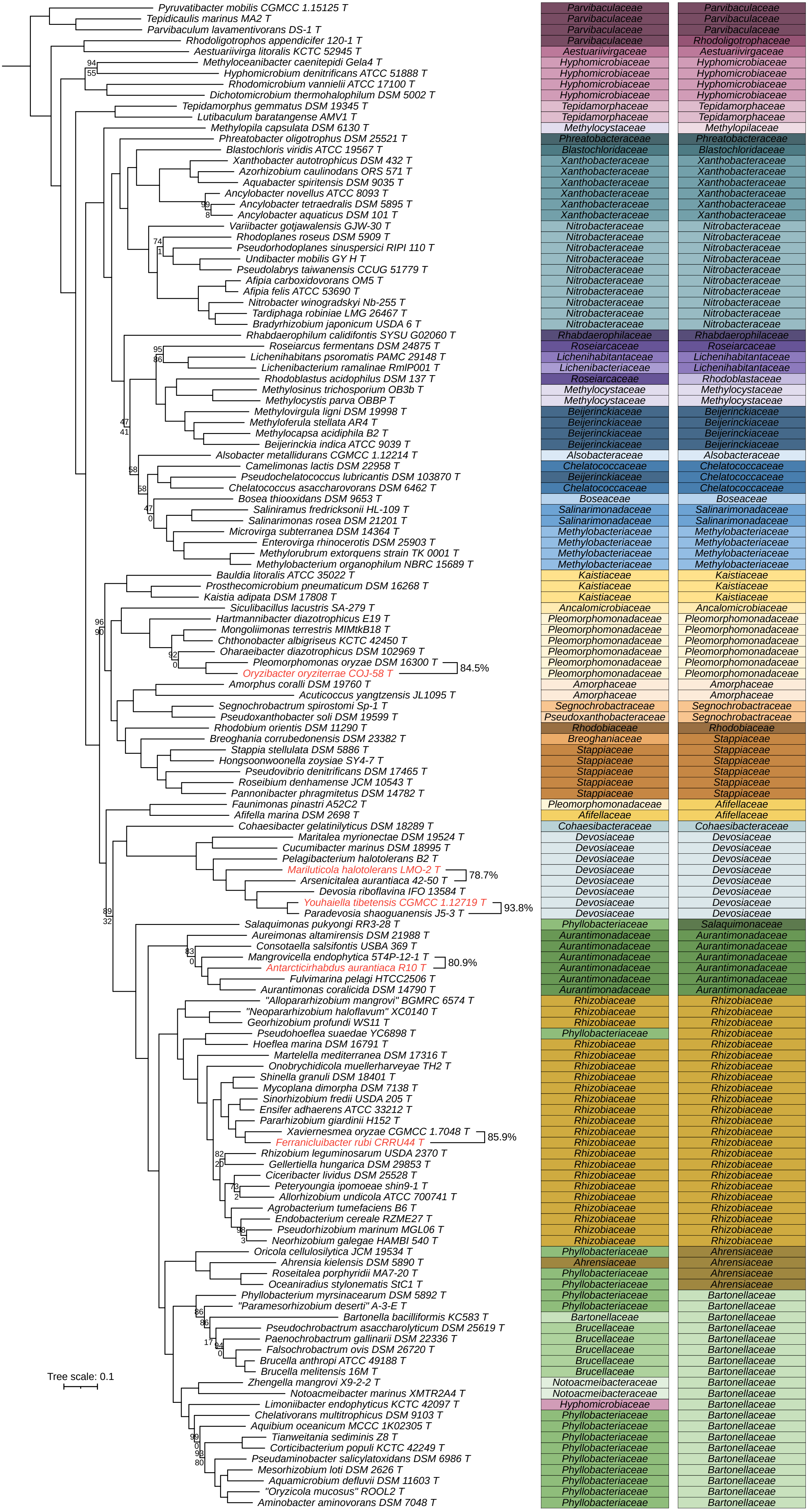

Figure S12. Validation of the proposed taxonomic framework. Our pipeline for assigning genera to families in the order *Hyphomicrobiales* was run on five genomes not included in our original analyses. Shown is the resulting maximum likelihood phylogeny, including 133 *Hyphomicrobiales* type strains from our original dataset (black font) and the five new genomes (red font). The phylogeny was inferred from the concatenated protein alignments encoded by the perc95_138 gene set, and five *Caulobacterales* type strains were included as the outgroup to root the phylogeny. The numbers on the nodes indicate the ultra-fast jackknife values using a 40% resampling rate (top numbers) and the SH-aLRT support values (bottom numbers), both calculated from 1000 replicates. Only values below 100 are shown. The scale bar represents the average number of amino acid substitutions per site. The numbers to the right of the phylogeny show the pairwise cpAAI values between a new genome and a neighbouring taxon; in all cases these numbers are greater than 75% indicating the strains belong to the same family. To the right of this are the current family assignments of each of the 138 *Hyphomicrobiales* type strains, followed to the right by the proposed family assignments of each strain.
